# GSK3β phosphorylation catalyzes the aggregation of tau into Alzheimer's disease-like filaments

**DOI:** 10.1073/pnas.2414176121

**Published:** 2024-12-18

**Authors:** Pijush Chakraborty, Alain Ibáñez de Opakua, Jeffrey A. Purslow, Simon A. Fromm, Debdeep Chatterjee, Milan Zachrdla, Shannon Zhuang, Sambhavi Puri, Benjamin Wolozin, Markus Zweckstetter

**Affiliations:** ^a^Department for NMR-based Structural Biology, Max Planck Institute for Multidisciplinary Sciences, Göttingen 37077, Germany; ^b^German Center for Neurodegenerative Diseases, Göttingen 37075, Germany; ^c^European Molecular Biology Laboratory Imaging Centre, European Molecular Biology Laboratory, Heidelberg 69117, Germany; ^d^Department of Pharmacology and Experimental Therapeutics, Boston University School of Medicine, Boston, MA 02118; ^e^Center for Neurophotonics, Boston University, Boston, MA 02215; ^f^Center for Systems Neuroscience, Boston University, Boston, MA 02215

**Keywords:** tau, Alzheimer's disease, phosphorylation, NMR, cryo-EM

## Abstract

The pathological deposition of proteins is a hallmark of several devastating neurodegenerative diseases. These pathological deposits comprise aggregates of proteins that adopt distinct structures named strains. However, the molecular factors responsible for the formation of distinct aggregate strains are unknown. Here, we show that the serine/threonine kinase GSK3β catalyzes the aggregation of the protein tau into Alzheimer’s disease (AD)-like filaments. We demonstrate that phosphorylation by GSK3β, but not by several other kinases, promotes the aggregation of full-length tau as well as enhances phase separation into gel-like condensate structures. Cryoelectron microscopy further reveals that the fibrils formed by GSK3β-phosphorylated tau adopt a fold comparable to that of paired helical filaments isolated from the brains of AD patients. Our results elucidate the intricate relationship between posttranslational modification and the formation of tau strains in neurodegenerative diseases.

## Significance

Alzheimer’s disease (AD) is characterized by the accumulation of neurofibrillary tangles within neurons, primarily composed of insoluble aggregates of tau protein. While tau hyperphosphorylation is a key factor in AD pathogenesis, the precise roles of different kinases remain unclear. This study examines how AD-associated kinases influence tau aggregation by phosphorylating specific residues. Notably, it identifies GSK3β as a critical kinase that accelerates tau aggregation and phase separation. Furthermore, the study highlights GSK3β phosphorylation as a significant molecular driver in the formation of AD-like filament structures, thereby highlighting the connection between posttranslational modification and tau strain formation.

More than 30 neurodegenerative diseases are characterized by the pathological aggregation of the microtubule-associated protein tau into insoluble deposits (1, 2). In Alzheimer’s disease (AD), tau deposition correlates with disease progression (3). Tau deposits in AD are composed of fibrils (4, 5). Tau is hyperphosphorylated in the diseased brain of AD and other tauopathies (3, 6). Phosphorylation has thus been suggested to modulate the formation of pathological tau aggregates and subsequent neuronal dysfunction (7). Additionally, posttranslational modifications, potentially together with so-far unknown cofactors, were suggested to drive the tau protein into distinct fibril structures (8, 9). These distinct tau fibril structures are named strains and are associated with different tauopathies (9–11). However, the molecular factors responsible for the formation of specific tau strains remain unknown.

Phosphorylation is the most abundant modification observed in tau that regulates the physiological activities of tau by modulating its binding to microtubules and other cellular compartments (12). Many serine/threonine kinases phosphorylate tau in the brain, including glycogen synthase kinase 3 (GSK3), cyclin-dependent kinase 5 (CDK5), microtubule affinity-regulating kinases (MARK), calmodulin-dependent protein kinase II (CaMKII), extracellular signal-regulated kinase (ERK), and cyclic AMP-dependent protein kinase (PKA)(12, 13). Furthermore, tyrosine kinases, such as FYN, SYK, and ABL, can phosphorylate tau’s tyrosine residues (12). Phosphorylation of tau inside the microtubule-binding domain reduces its affinity toward the negatively charged microtubules (14, 15). Under pathological conditions, approximately 40 to 50 out of 85 potential phosphorylation sites in tau have been identified to undergo phosphorylation (16).

A growing number of studies suggest that biomolecular condensation may contribute to the pathogenesis of neurodegenerative diseases by acting as an intermediate or transition state between the monomeric protein and its pathological aggregated state (17, 18). Consistent with this hypothesis, condensate formation can enhance the aggregation of intrinsically disordered proteins into fibrils (19, 20). Posttranslational modifications including phosphorylation modulate biomolecular condensation either by promoting the phase separation of intrinsically disordered proteins or by inhibiting it (21). For example, phosphorylation in the proline-rich domain of tau promotes the protein’s phase separation, while acetylation blocks tau phase separation (22–24). However, little is known about the relationship between phosphorylation, tau condensation and aggregation, and their impact on the formation of distinct tau fibril structures in neurodegenerative diseases.

## Results

### Kinase-Specific Phosphorylation Patterns of Tau.

**Fig. 1.**
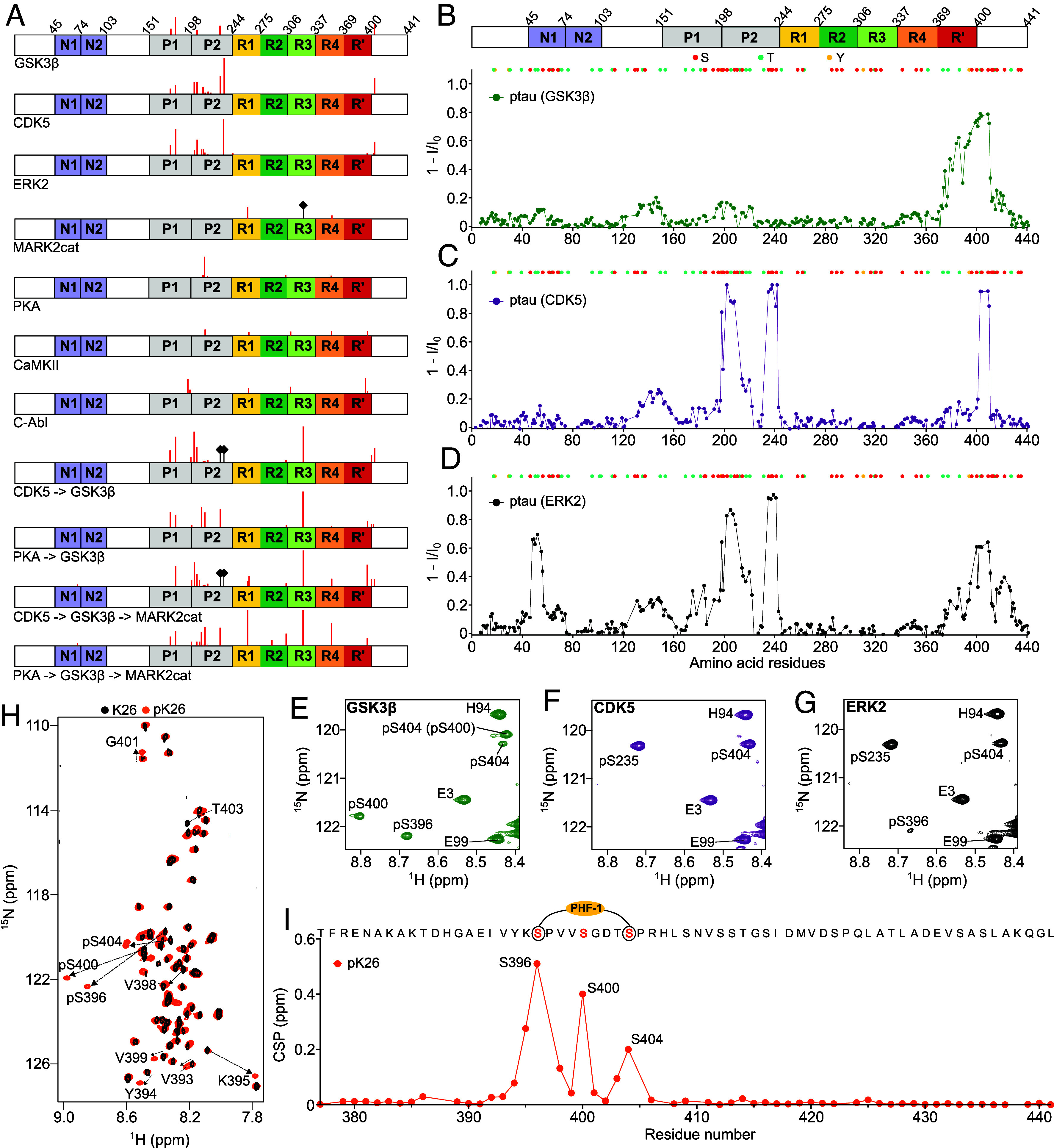
Kinase-specific phosphorylation patterns of tau. (*A*) Domain diagram of full-length tau. The residues phosphorylated by different kinases and detected by mass spectrometry are indicated with red bars. The lengths of the red ticks are adjusted based on the percentage of phosphorylation calculated by dividing the number of peptides detected containing a particular residue by the total number of peptides detected containing the same residue. The residue-specific percentage of phosphorylation can be found in Dataset S1. The black diamond-headed bars refer to previously reported phosphorylation sites that were not detected due to the absence of peptides in the mass spectrometry experiment. (*B*–*D*) Residue-specific intensity changes observed in 2D ^1^H-^15^N HSQC spectra of tau upon phosphorylation by GSK3β (*B*), CDK5 (*C*) and ERK2 (*D*). I and I_0_ are the cross-peak intensities of the phosphorylated and unmodified tau, respectively. The location of serine, threonine, and tyrosine residues is indicated above. (*E*–*G*) Signals of the cross-peaks of phosphorylated serine residues in the ^1^H-^15^N HSQC spectra of phosphorylated tau appeared due to the phosphorylation by GSK3b (*E*), CDK5 (*F*), and ERK2 (*G*). pS404 (pS400) shown in (*E*) denotes the peak of pS404 shifted due to the presence of pS400. (*H*) ^1^H-^15^N HSQC spectra of either unmodified (black) or GSK3β-phosphorylated (orange) C-terminal fragment of tau comprising residues 369 to 441 (referred to as K26). The cross-peaks of the phosphorylated residues and the residues shifted due to nearby phosphorylation are shown in the spectrum. (*I*) Residue-specific chemical-shift perturbation (CSP) observed in the ^1^H-^15^N HSQC spectra of K26 upon phosphorylation by GSK3β. The PHF-1 epitope is marked in the amino acid sequence displayed above.

To identify the tau phosphorylation patterns of different kinases, we performed individual phosphorylation reactions of the full-length 2N4R isoform of tau (441 residues; further referred to as tau) in vitro. For the phosphorylation reactions, we selected the six serine/threonine kinases GSK3β, CDK5, ERK2, MARK2cat, PKA, and CaMKII, as well as the tyrosine kinase C-Abl (Fig. 1*A*). To assess primed phosphorylation, we also phosphorylated tau with two or three kinases in a step-wise manner: CDK5 followed by GSK3β, PKA followed by GSK3β, CDK5 followed by GSK3β and MARK2cat, as well as PKA followed by GSK3β and subsequently MARK2cat (Fig. 1*A*). To determine the phosphorylation patterns of tau, the phosphorylated samples were loaded in an SDS-PAGE gel (*SI Appendix*, Fig. S1). Subsequently, the tau bands observed in the SDS-PAGE gel were analyzed by mass spectrometry. The extent of phosphorylation for each residue was determined by the ratio between the number of detected peptides containing a particular phosphorylated residue and the total number of peptides detected containing the same residue (Dataset S1).

The analysis provided insights into the specific tau phosphorylation patterns of the seven kinases. In the case of GSK3β, we detected phosphorylation of S404, as well as phosphorylation of some residues in the proline-rich domain (T175, T181, T205, and T231) (Fig. 1*A*). However, detection of a very small number of peptides from tau’s C terminus precluded confirmation of phosphorylation of S396 and S400, the two other serine residues in the C terminus of tau that were previously shown to be also phosphorylated by GSK3β (25). In the case of CDK5, we detected phosphorylation of the C-terminal site S404, as well as phosphorylation of T175, T181, S202, T205, T212, S214, T217, T220, T231, and S235 in the proline-rich domain (Fig. 1*A*). The kinase ERK2 phosphorylated S396, T403, and S404 in the C terminus of tau, as well as other sites, including T175, T181, S202, T205, S210, T212, S214, T217, S235, and T245.

The other three tested kinases (PKA, MARK2cat, and CaMKII) also phosphorylated residues in the aggregation-prone repeat-domain of tau (Fig. 1*A*), but the sites differed from the specific sites phosphorylated by GSK3β, CDK5, and ERK2. For example, a phosphorylation reaction of 15 min in the presence of PKA results in the phosphorylation of S305 and S356 in repeats 2 and 4, respectively, together with phosphorylation of S214. Similarly, the catalytic domain of the kinase MARK2 (MARK2cat) phosphorylated S262 and S356. The phosphorylation at S324 by MARK2cat (26) was not detected because of the absence of peptides from that region. In agreement with previous reports (25), CaMKII phosphorylated S214, T263, and S356 of tau. A small number of peptides with phosphorylated Y310 and Y394 were also detected after CaMKII phosphorylation (Fig. 1*A*). The tyrosine kinase C-Abl phosphorylated Y197, Y310, and Y394 (phosphorylation of Y18 and Y29 was not observed due to the detection of few peptides from the N terminus of tau). Sequential phosphorylation of tau by two or three kinases leads to an abundant phosphorylation in both the proline-rich domain and the repeat domain, with most peptides detected for phosphorylated S324 (Fig. 1*A*).

### GSK3β Most Efficiently Phosphorylates Tau’s PHF-1 Epitope.

Mass spectrometry is extremely sensitive to detect even very small amounts of phosphorylation. However, differential ionization of peptides from different regions of the protein limits the precise quantification of residue-specific phosphorylation levels. On the other hand, the residue-specific extent of phosphorylation can be accurately determined by NMR spectroscopy. To gain further insights into the sites and degree of phosphorylation catalyzed by the serine/threonine kinases GSK3β, CDK5, and ERK2, we used NMR spectroscopy. We phosphorylated ^15^N-labeled 2N4R tau in the presence of either kinase and recorded two-dimensional (2D) ^1^H-^15^N HSQC NMR spectra of the phosphorylated proteins. Comparison of the cross-peaks intensities in the phosphorylated samples to the unmodified sample revealed that the three kinases efficiently phosphorylated residues in the C-terminal domain of tau (Fig. 1 *B*–*D*). The detection of additional cross-peaks of the phosphorylated residues confirmed that GSK3β efficiently phosphorylates S396 and S404, i.e., the two residues that comprise the PHF-1 epitope (27), as well as S400 (Fig. 1*E* and *SI Appendix*, Fig. S2). The extent of phosphorylation of S404 (~75%) and S400 (~57%) by GSK3β was calculated by comparing the intensity of the phosphorylated cross-peak to the unmodified cross-peak. On the other hand, we were unable to precisely calculate the extent of phosphorylation of S396 due to signal overlap of unmodified S396 with T231. However, based on the intensity loss of nearby residues, we expect ~60% phosphorylation of S396. Notably, the NMR-based intensity analysis also shows that the other sites (T175, T181, T205, and T231) in the proline-rich domain of tau, for which phosphorylation was detected by mass spectrometry (Fig. 1*A*), are only very weakly phosphorylated by GSK3β. As these sites are weakly phosphorylated by GSK3β, we also did not detect signals of the phosphorylated peaks of these residues. GSK3β thus most efficiently phosphorylates the PHF-1 epitope in the C terminus of tau.

We further confirmed phosphorylation of S396, S400, and S404 by GSK3β-catalyzed phosphorylation of a C-terminal fragment of tau comprising residues 369 to 441 (referred to as K26) and subsequent analysis by NMR. The analysis revealed that GSK3β indeed phosphorylates S396, S400, and S404 in the C-terminal domain (Fig. 1 *H* and *I* and *SI Appendix*, Fig. S3), where the extent of phosphorylation were determined to be approximately 56%, 59%, and 81%, respectively. The extent of phosphorylation is representative of a single experiment performed on a single batch of protein.

The NMR analysis also showed that ERK2 phosphorylated both S404 and S396 but not S400 (Fig. 1*G* and *SI Appendix*, Fig. S2). The extent of phosphorylation of S404 is ~55%. Notably, the cross-peak of phosphorylated S396 was weaker than in case of GSK3β phosphorylation (Fig. 1*G*), suggesting that ERK2 is less efficient in phosphorylating S396 when compared to GSK3β. Although we could not precisely calculate the extent of phosphorylation of S396 due to spectral overlap, based on the intensity loss of the neighboring residues we expect ~15% phosphorylation. In the case of CDK5 phosphorylation, we did not detect a cross-peak of phosphorylated S396, indicating that CDK5 only phosphorylates S404 in the C-terminal domain of tau (Fig. 1*F* and *SI Appendix*, Fig. S2). The extent of phosphorylation of S404 is ~93% by CDK5.

Besides phosphorylation at the C terminus, CDK5 and ERK2 (GSK3β to a lesser degree) effectively phosphorylated residues in the proline-rich domain of tau (Fig. 1 *B*–*D*), broadly in agreement with the results from mass spectrometry (Fig. 1*A*). The ERK2 kinase also phosphorylated some residues [most likely T50 (28)] in the N-terminal regions between residue 40 and 80 (Fig. 1*D*). The phosphorylation of these residues were not detected by mass spectrometry, because of the detection of very few peptides from the N terminus of tau. The combined data show that GSK3β and ERK2, but not the other tested kinases, phosphorylate the tau epitope (pS396 and pS404) that is recognized by the monoclonal antibody PHF-1 (27).

### GSK3β Phosphorylation Accelerates Tau Fibrilization.

**Fig. 2.**
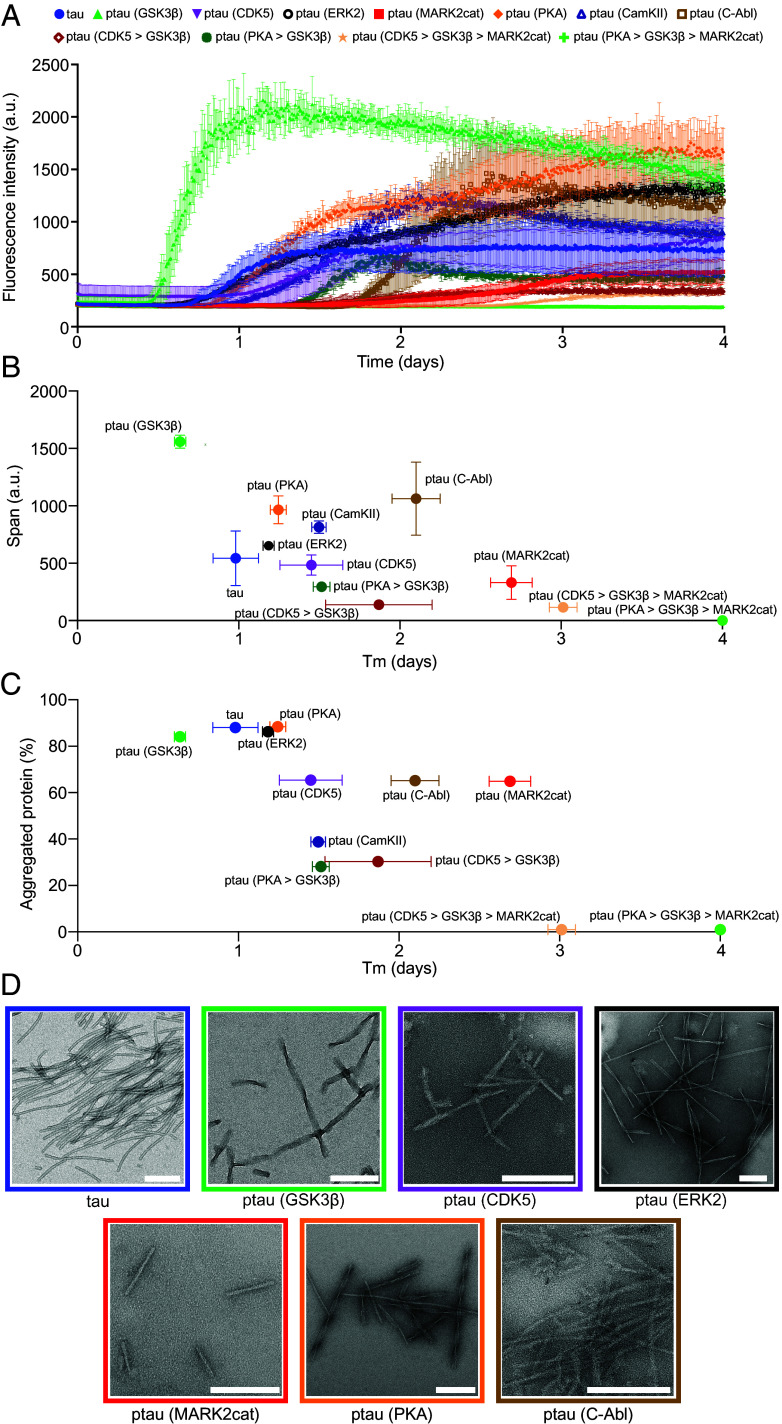
Acceleration of tau aggregation by GSK3β phosphorylation. (*A*) Aggregation kinetics of 25 µM unmodified tau and tau phosphorylated by different kinases. Error bars represent the std of three independently aggregated samples. (*B*) ThT-intensity span vs. half-time of aggregation (Tm) of unmodified and phosphorylated tau proteins. Error bars represent the std of three independently aggregated samples. (*C*) Amount of protein aggregated vs. half-time of aggregation (Tm) of unmodified and phosphorylated tau proteins. The amount of aggregated protein was calculated by comparing the intensity of the supernatant (SN) band (after pelleting down the fibrils) to the tau monomer band as shown in *SI Appendix*, Fig. S5. Error bars represent the std of the half-time of aggregation of three independently aggregated samples. (*D*) Negative-stain EM of fibrils formed by unmodified and different phosphorylated tau samples. (Scale bars, 200 nm.)

We next asked whether the distinct phosphorylation patterns imprinted by different kinases differentially modulate the aggregation of tau into insoluble deposits. To gain insight into this question, we aggregated the unmodified and phosphorylated tau proteins using our previously developed cofactor-free in vitro aggregation assay (29). We found distinct phosphorylation-specific changes in tau fibril formation: only GSK3β-phosphorylated tau aggregated faster than the unmodified protein (Fig. 2 *A* and *B* and *SI Appendix*, Fig. S4). Other tau proteins phosphorylated by a single kinase displayed slower aggregation when compared to unmodified tau. ERK2-phosphorylated tau was most similar to unmodified tau in its aggregation kinetics, followed by PKA-, CDK5-, and CamKII-phosphorylated tau (Fig. 2 *A* and *B* and *SI Appendix*, Fig. S4). C-Abl-phosphorylated tau displayed even slower aggregation kinetics, but tau aggregation was most impaired when phosphorylated by the catalytic domain of MARK2. Interestingly, stepwise phosphorylation by two kinases delayed tau aggregation when compared to phosphorylation by the respective individual kinases. Phosphorylation by two kinases and additionally by MARK2 largely blocked tau fibrillization during the time of incubation (Fig. 2 *A* and *B* and *SI Appendix*, Fig. S4). Thus, GSK3β phosphorylation selectively accelerates tau fibrilization.

In addition to phosphorylation-induced changes in the aggregation kinetics, we observed different final fluorescence intensities of the amyloid-specific dye thioflavin-T (ThT) for tau proteins phosphorylated by different kinases (Fig. 2 *A* and *B* and *SI Appendix*, Fig. S4). Most of the phosphorylated tau proteins displayed higher ThT intensity at the end of aggregation when compared to the unmodified protein, apart from tau proteins that were phosphorylated by multiple kinases (Fig. 2 *A* and *B*).

To evaluate whether the higher ThT intensity is caused by an increased amount of aggregated protein, we centrifuged the aggregated samples at the end of the incubation period. Subsequently, the amount of residual protein was analyzed by loading the supernatants into an SDS-PAGE gel (*SI Appendix*, Fig. S5). Comparison of the band intensity of the supernatants with the unmodified monomeric protein revealed that a similar amount of protein was aggregated for unmodified tau and tau phosphorylated by the kinases GSK3β, ERK2 and PKA (Fig. 2*C*). Approximately 80 to 90% of the monomeric proteins were aggregated into insoluble deposits (Fig. 2*C*). In contrast, phosphorylation of tau by CDK5, C-Abl, and MARK2cat decreased the amount of aggregated protein to ~60% (Fig. 2*C*). The amount of aggregated protein further decreased when tau was phosphorylated by CamKII or in the case of sequential phosphorylation with two kinases. Finally, the sequential phosphorylation of tau in the presence of three kinases, either PKA or CDK5 followed by GSK3β and MARK2cat, abolished tau fibrillization. Thus, the higher ThT intensity observed for most of the fibrils formed by phosphorylated tau proteins is likely caused by differences in ThT binding, indicating the formation of distinct amyloid fibril structures by phosphorylated tau proteins when compared to unmodified tau.

We then recorded the negative-stain EM images of unmodified and different phosphorylated tau samples (Fig. 2*D*). In the EM images of GSK3β, ERK2, and PKA-phosphorylated tau fibrils, we observed separated fibrils with a pronounced twist (Fig. 2*D*). These twisted fibrils differ from the fibrils formed by unmodified, CDK5, MARK2cat, and C-Abl-phosphorylated tau which were mostly straight (Fig. 2*D*). The EM images of CamKII-phosphorylated tau fibrils and fibrils formed by tau phosphorylated with multiple kinases could not be recorded due to the presence of few aggregates in these samples.

The above data indicate that only GSK3β phosphorylation accelerates tau fibril formation when compared to the unmodified protein (Fig. 2 *A*–*C*) and forms morphologically distinct fibrils compared to the unmodified tau (Fig. 2*D*). To validate that the fibrils formed by GSK3β-phosphorylated tau are indeed formed by the phosphorylated protein and not the minor population of unmodified tau present in the sample, we performed western blot analysis of both the GSK3β-phosphorylated and unmodified tau fibrils (*SI Appendix*, Fig. S6). The GSK3β-phosphorylated and unmodified tau fibrils were pelleted down and the pellets were separated by SDS-PAGE (*SI Appendix*, Fig. S6*A*) and identified with the anti-phospho-tau (S396) antibody (*SI Appendix*, Fig. S6*B*). The anti-phospho-tau (S396) antibody selectively detected the GSK3β-phosphorylated tau fibrils and not the fibrils formed by unmodified tau confirming that the GSK3β-phosphorylated tau fibrils are indeed formed by the phosphorylated protein.

### Phosphorylation at the PHF-1 Epitope Catalyzes the Condensation of Tau.

**Fig. 3.**
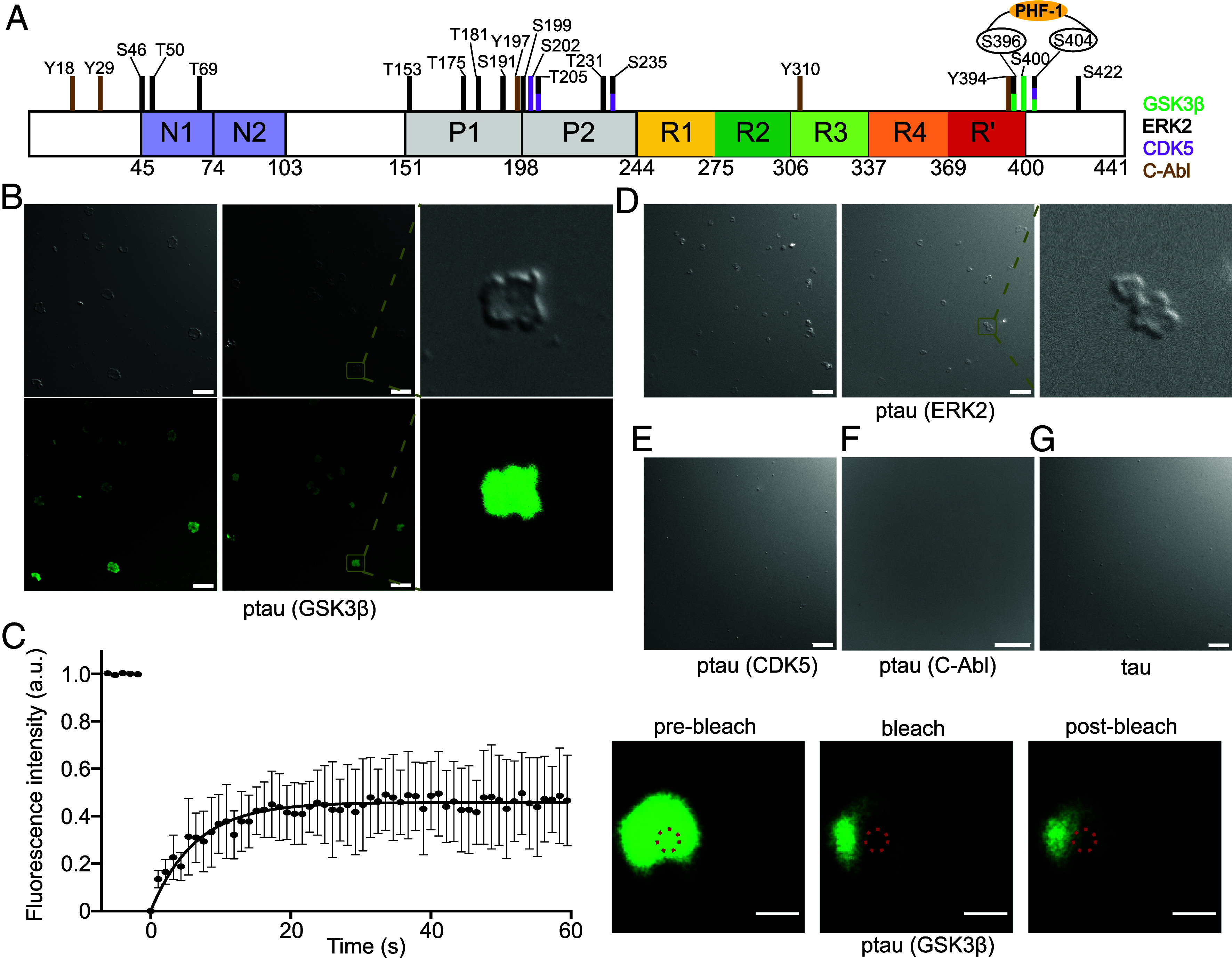
GSK3β phosphorylation promotes tau condensation. (*A*) Domain diagram of 2N4R tau. Residues that undergo phosphorylation in the presence of GSK3β, ERK2, CDK5, and C-Abl are marked with green, black, purple, and dark-yellow colored bars, respectively. S396 and S404, which are phosphorylated by GSK3β, form the epitope that is recognized by the phosphorylation-specific antibody PHF-1. (*B*) DIC and fluorescence microscopy of the condensates formed by 25 µM GSK3β-phosphorylated tau at room temperature in the aggregation assay buffer (25 mM HEPES, 10 mM KCl, 5 mM MgCl_2_, 3 mM TCEP, and 0.01% NaN_3_, pH 7.2). A zoomed-in view of the condensate is shown to the *Right*. The protein was labeled with Alexa Fluor 488 dye. Micrographs are representative of three independent biological replicates. (Scale bar, 10 µm.) (*C*) Fluorescence recovery after photobleaching (FRAP) experiment of the condensates formed by GSK3β-phosphorylated tau shown in (*B*). The protein was labeled with Alexa Fluor 488 dye. Error bars represent std of averaged three curves for each time point. Representative micrographs of the condensate before bleaching, after bleaching, and at the end of recovery are displayed to the *Right*. (Scale bar, 1 µm.) (*D*) DIC microscopy of the condensates formed by 25 µM ERK2-phosphorylated tau at room temperature in the aggregation assay buffer. A zoomed-in view of the condensate is shown to the *Right*. Micrographs are representative of three independent biological replicates. (Scale bar, 10 µm.) (*E*–*G*) DIC microscopy of 25 µM CDK5-phosphorylated tau (*E*), C-Abl-phosphorylated tau (*F*), and unmodified tau (*G*) at room temperature in the aggregation assay buffer. Micrographs are representative of three independent biological replicates. (Scale bar, 10 µm.)

To understand whether the accelerated aggregation of GSK3β-phosphorylated tau is linked to the formation of condensates, we imaged the GSK3β-phosphorylated tau samples by differential interference contrast (DIC) microscopy prior to starting the aggregation assay. Under this condition, i.e., after incubation for 10 min at room temperature in the aggregation assay buffer, we observed the formation of condensates in the GSK3β-phosphorylated tau sample (Fig. 3*B*). Fluorescence microscopy confirmed that these condensates are indeed formed by GSK3β-phosphorylated tau (Fig. 3*B*). In contrast, the unmodified tau did not form visible condensates in these conditions (Fig. 3*G*).

Closer inspection of the DIC images showed that the condensates formed by GSK3β-phosphorylated tau did not possess the typical spherical shape of liquid droplets but displayed a more “squeezed” appearance. These condensates were also dimly stained by the amyloid-binding dye Thioflavin-T (*SI Appendix*, Fig. S7). To gain insight into the diffusive properties of these condensates, we used fluorescence recovery after photobleaching. Although part of the fluorescence recovered rapidly in a few seconds, an immobile fraction of 50 to 80% remained (Fig. 3*C*). This suggests that the material properties inside the GSK3β-phosphorylated tau condensates differ from liquid droplets and contain both more diffusive and gel-like tau protein.

The major GSK3β-induced phosphorylation sites in tau are located at the C terminus (S396, S400, and S404) (Figs. 1 *B* and *E* and 3*A*). Similar to GSK3β, ERK2 (S396 and S404), and CDK5 (S404) also phosphorylate tau at the C terminus (Figs. 1 *C*, *D*, *F*, and *G* and 3*A*). To elucidate the relationship between phosphorylation site and condensate formation, we imaged ERK2-phosphorylated as well as CDK5-phosphorylated tau with DIC microscopy prior to starting the aggregation assay (Fig. 3 *D* and *E*). In the aggregation assay buffer, we observed that ERK2-phosphorylated tau forms condensates similar to that of GSK3β-phosphorylated tau, but not CDK5-phosphorylated tau (Fig. 3 *D* and *E*). As an additional control, we asked whether other types of phosphorylation may promote condensate formation. However, tau phosphorylated by the tyrosine kinase C-Abl, which phosphorylates multiple of the tyrosine residues of tau including Y394 at the C terminus, did not form condensates under the same conditions (Fig. 3*F*). This suggests that phosphorylation at only S404 is not sufficient, but it requires phosphorylation of both S396 and S404, that is the PHF-1 epitope, to promote tau condensation.

### Tau Droplets Mature into Gel-Like Structures Upon GSK3β Phosphorylation.

**Fig. 4.**
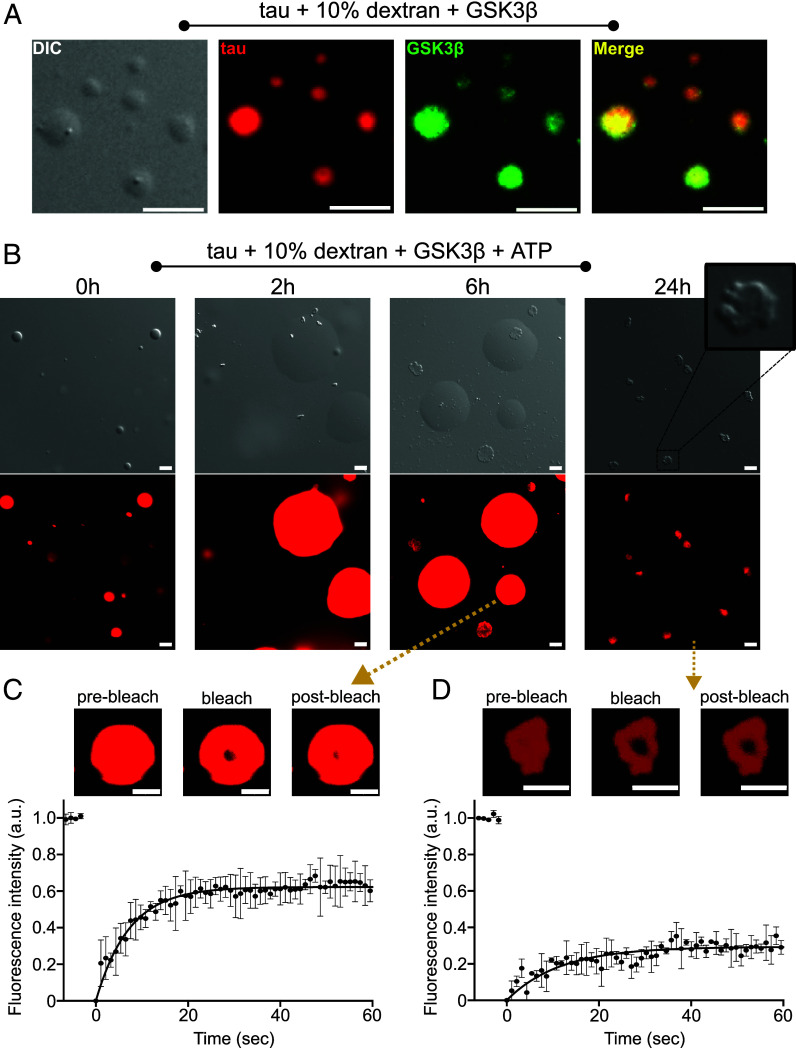
Maturation of tau droplets into gel-like structures upon GSK3β phosphorylation. (*A*) DIC and fluorescence microscopy of tau droplets induced by the addition of 10% dextran at room temperature in 25 mM HEPES, 10 mM KCl, 5 mM MgCl_2_, pH 7.2 buffer. Fluorescently labeled GSK3β partitioned into the droplets. Tau and GSK3β were labeled with Alexa Fluor 594 and Alexa Fluor 488 dye, respectively. Micrographs are representative of three independent biological replicates. (Scale bar, 5 µm.) (*B*) DIC and fluorescence microscopy of the tau droplets induced by the addition of 10% dextran at room temperature in 25 mM HEPES, 10 mM KCl, 5 mM MgCl_2_, pH 7.2 buffer in the presence of 0.02 mg/mL unlabeled GSK3β, and 1 mM ATP. The sample was incubated for 24 h. A zoomed-in view of the condensate formed after 24 h is shown. Tau was labeled with Alexa Fluor 594 dye. Micrographs are representative of three independent biological replicates. (Scale bar, 5 µm.) (*C*) FRAP experiment of the larger fused condensates of tau in the presence of GSK3β and ATP after incubation for 6 h. Tau was labeled with Alexa Fluor 594 dye. The yellow arrow indicates that the FRAP experiments were performed on the larger condensates formed after incubation for 6 h. Error bars represent std of averaged three curves for each time point. Representative micrographs of the condensate before bleaching, after bleaching, and at the end of recovery are displayed on the *Top*. (Scale bar, 5 µm.) (*D*) FRAP experiment of the gel-like condensates of tau in the presence of GSK3β and ATP after incubation for 1 d. Tau was labeled with Alexa Fluor 594 dye. The yellow arrow indicates that the FRAP experiments were performed on the gel-like condensates formed after incubation for 1 d. Error bars represent std of averaged three curves for each time point. Representative micrographs of the condensate before bleaching, after bleaching, and at the end of recovery are displayed on the *Top*. (Scale bar, 5 µm.)

Crowding agents promote the liquid–liquid phase separation (LLPS) of unmodified tau (22, 30, 31). In agreement with these reports, we observed the formation of spherical, highly mobile droplets of unmodified tau in the presence of 10% dextran (Fig. 4*A*). We then added fluorescently labeled GSK3β to the preformed tau droplets and observed that the kinase rapidly enters the droplets (Fig. 4*A*). GSK3β thus concentrates inside the tau droplets where it might phosphorylate tau.

To assess whether GSK3β phosphorylates tau and changes the morphology of tau droplets, we repeated the experiment but now added unlabeled GSK3β as well as ATP to the preformed tau droplets. During the first 2 h of incubation, we observed mobile tau droplets that fused and formed larger droplets (Fig. 4*B*). After 6 h of incubation, we again observed the larger mobile tau droplets, but additionally some smaller squeezed-like condensates appeared (Fig. 4*B*). After 1 d of incubation, we observed the presence of only such gel-like condensates in the solution (Fig. 4*B*). They were morphologically similar to the condensates formed by the GSK3β-phosphorylated tau in the absence of a crowding agent (Fig. 3*B*). In contrast, we did not observe such gel-like condensates upon the incubation of tau droplets only in the presence of GSK3β, i.e., under nonphosphorylating conditions without ATP (*SI Appendix*, Fig. S8).

Mass spectrometry confirmed that the tau sample was indeed phosphorylated under the condition of LLPS (*SI Appendix*, Fig. S9). Notably, the total degree of phosphorylation was higher when phosphorylation occurred under the conditions of LLPS when compared to GSK3β-induced phosphorylation in the dispersed phase (*SI Appendix*, Fig. S9), in agreement with the acceleration of phosphorylation in condensed states of tau (32).

To get further insight into the dynamics inside the tau condensates, we performed FRAP experiments. Photobleaching the larger tau condensates formed with GSK3β but without ATP, i.e., in nonphosphorylating conditions, after incubation for 6 h resulted in an immobile fraction of ~20% (*SI Appendix*, Fig. S8*B*). A prolonged incubation of 1 d resulted in an immobile fraction between 30% and 40% (*SI Appendix*, Fig. S8*C*). In contrast, photobleaching the larger tau condensates formed in the presence of both GSK3β and ATP after incubation for 6 h revealed the presence of immobile fractions of ~40% (Fig. 4*C*). Additionally, photobleaching the gel-like condensates, which formed only in the presence of both GSK3β and ATP after an incubation of 1 d, resulted in an immobile fraction of ~80% (Fig. 4*D*). The ~80% immobile fraction is at the upper end of immobile fractions observed in the condensates formed by GSK3β-phosphorylated tau in the aggregation assay buffer without crowding agents (Fig. 3*C*). This may result from a longer incubation time, different buffer conditions, or the presence of dextran in the current experiments. Taken together, the data show that tau droplets mature into gel-like structures upon GSK3β phosphorylation.

### GSK3β-Phosphorylated Tau Folds into AD-Like Filaments.

The above experiments show that the specific phosphorylation of the PHF-1 epitope by GSK3β promotes the condensation of tau into gel-like structures that might facilitate its conversion into amyloid fibrils under conditions of agitation. To characterize the stable core region of the fibrils formed by GSK3β-phosphorylated tau, we combined pronase digestion with mass spectrometry. We first digested GSK3β-phosphorylated tau fibrils by pronase, centrifuged the sample, and then loaded the pellet on an SDS-PAGE (*SI Appendix*, Fig. S10*A*). Subsequently, the tau band at ~12 kDa was cut and digested by trypsin, followed by detection of the peptides using an ESI mass spectrometer. Analysis of the number of detected peptides as a function of sequence revealed peptides belonging to residues ~280 to ~400 (*SI Appendix*, Fig. S10*B*). The pronase-resistant core of GSK3β-phosphorylated tau fibrils thus comprises residues ~280 to ~400, which is comparable to the core of tau fibrils extracted by sarkosyl from the brain tissues of AD patients (33).

**Fig. 5.**
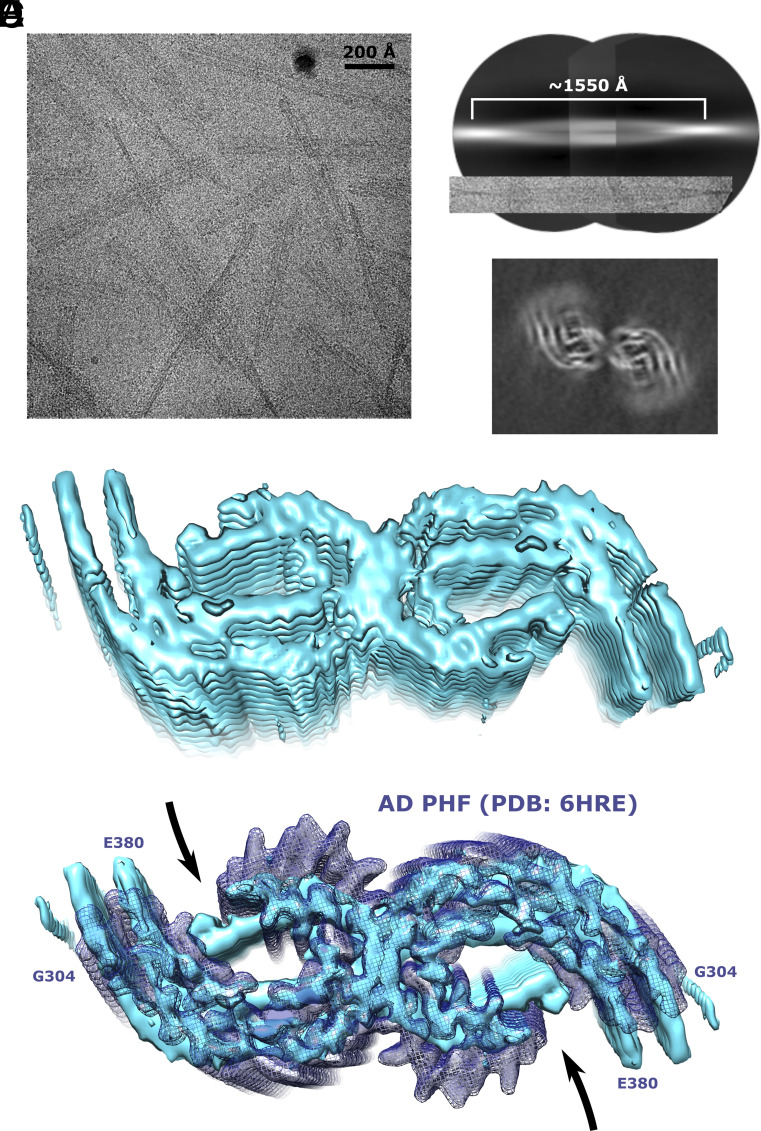
Cryo-EM of GSK3β-phosphorylated tau fibrils. (*A*) Cryoelectron micrograph of GSK3β-phosphorylated tau fibrils. (*B*) Reconstruction of a GSK3β phosphorylated tau fibril from low-resolution and big box 2D classes for cross-over estimation. (*C*) Cross-section of the cryo-EM map of the GSK3β-phosphorylated tau fibril after 3D refinement. (*D*) Cryo-EM density map of GSK3β phosphorylated tau fibrils. (*E*) Cryo-EM density map of GSK3β-phosphorylated tau fibrils (cyan surface) compared with the paired helical filament (PHF) from sporadic AD brain (dark blue isomesh; PDB id: 6HRE).

To understand the structure of GSK3β-phosphorylated tau fibrils at higher resolution, we utilized cryo-EM. The cryo-EM images of the GSK3β-phosphorylated tau revealed the presence of both straight and twisted filaments (Fig. 5*A*). The average cross-over distance of the twisted fibrils was ~1,550 Å (Fig. 5*B*). 3D reconstructions of the twisted filaments were calculated to an overall resolution of ~5 Å using helical reconstruction in RELION (34, 35) (*SI Appendix*, Fig. S11 and Table S1).

The GSK3β-phosphorylated tau fibrils are composed of two protofilaments with closed C-shaped subunits (Fig. 5 *C* and *D*). The protofilaments are related by C2 symmetry and the successive rungs of β-sheets in each protofilament are related through helical symmetry with a rise of 4.75 Å and twist of approximately −0.55° (*SI Appendix*, Fig. S11*A* and Table S1).

The cryo-EM analysis shows that the structure adopted by the GSK3β-phosphorylated tau fibrils is similar to that of PHF derived from the brain of AD patients (Fig. 5*E*). Indeed, the location of the rigid core is comparable, the individual filaments fold into a C-shaped conformation, and the two filaments have a similar relative arrangement (Fig. 5*E*). While the current resolution does not allow further analysis, the protofilaments of the in vitro aggregated GSK3β-phosphorylated tau fibrils may adopt a more closed C-shaped conformation when compared to the AD PHFs (Fig. 5*E*), which are related by a pseudo 2-1 screw symmetry (33, 36). The GSK3β-phosphorylated tau fibrils may thus be more similar to the PHFs extracted from the extracellular vesicles isolated from the brains of people with AD (*SI Appendix*, Fig. S12).

## Discussion

The proline-directed serine/threonine kinase GSK3β is closely associated with the pathological phosphorylation of tau in AD (37–39). In AD patients, GSK3β colocalizes with neurofibrillary tangles (40, 41) and concentrates in the frontal cortex of AD brains (38). In animal models, increased GSK3β activity leads to tau hyperphosphorylation and downstream neurodegeneration (37). Accordingly, an isoform-selective decrease of GSK3β reduces synaptic tau phosphorylation, transcellular spreading, and aggregation (39). Here, we showed that GSK3β most efficiently phosphorylates the PHF-1 epitope of tau, catalyzes the phase separation of tau into gel-like condensates, and selectively accelerates in vitro aggregation of tau into fibrils that adopt a fold similar to that found in PHFs extracted from AD patient brains.

Using our recently described cofactor-free aggregation assay of full-length tau, we showed that GSK3β but none of the other five tested kinases, nor the combinations of two or three kinases, promote the aggregation of tau into fibrils (Fig. 2). Using a combination of mass spectrometry and NMR spectroscopy, we linked the accelerated aggregation of GSK3β-phosphorylated tau to the ability of GSK3β to efficiently phosphorylate S396 and S404 of tau, which are recognized by the monoclonal antibody PHF-I (Fig. 1). While the kinase ERK2 also phosphorylates both S396 and S404, the degree of S396 phosphorylation was lower when compared to GSK3β phosphorylation. The efficient phosphorylation of S396 and the additional phosphorylation of S400, which is only phosphorylated by GSK3β, may contribute to the GSK3β-specific acceleration of tau aggregation. The three serine residues at the C terminus (S396, S400, and S404) that are phosphorylated by GSK3β, are also abnormally hyperphosphorylated in the brain of AD patients (42). Notably, phosphorylation at the N terminus and in particular in the repeat domain delayed tau aggregation (Fig. 1). Our study also shows that GSK3β without priming by another kinase only weakly phosphorylates residues T181 and T231, sites in the proline-rich domain that are likely phosphorylated early in the disease process. This indicates that it may be important to further investigate priming of GSK3β phosphorylation by a variety of kinases and its impact on tau condensation and aggregation.

Our data further reveal that under the conditions of aggregation, tau phosphorylated by GSK3β (without priming by other kinases) forms condensates with gel-like properties. In contrast, unmodified tau did not form condensates under the same conditions (Fig. 3). The condition of the condensate formation may increase the kinetics of aggregation because the high tau concentration inside the condensates may lead to faster aggregation on the surface of the condensate (19, 20). Additionally, we found that phosphorylation by GSK3β is stronger in conditions of phase separation when compared to the dispersed phase. Phosphorylation by GSK3β and tau condensation may thus reinforce each other. The connection between tau condensation and accelerated aggregation was further supported by our observation that other types of phosphorylated tau protein exhibit delayed aggregation when compared to unmodified tau and do not form condensates (Fig. 3 *E*–*G*). Phosphorylation of tau by ERK2 elicited condensate formation without enhanced aggregation, which contrasts with observations for the other kinase/tau pairings (Figs. 2 and 3). These data point to additional mechanisms—in particular to the regulatory role of phosphorylation in the proline-rich domain—that differentially regulate tau condensation and aggregation.

In brain-derived extracts from AD patients, filaments comprising only two C-shaped protofilaments of tau have so far been observed (33, 36) (Fig. 5*E*). Additionally, a truncated fragment of 4R tau comprising residues 297 to 391 was recently shown to aggregate in vitro into a fibril structure comprising two protofilaments that is highly similar to that of ex vivo PHFs (43). The same study also reported that another C-terminal pseudophosphorylated 4R tau fragment (S396D, S400D, T403D, and S404D) comprising residues from 297 to 441 adopts a single protofilament with the AD fold (43, 44). Although this study reported the in vitro aggregation of a 4R tau fragment into the AD fold comprising two protofilaments, the tau species that are deposited in the brains of AD patients are predominantly the full-length isoforms of both 4R and 3R tau (7, 43, 45). Here, we showed that the full-length 4R isoform of tau phosphorylated at the C terminus by the AD-associated kinase GSK3β aggregates in vitro into filaments that are composed of two C-shaped protofilaments with a fold that is similar to that of ex vivo PHFs (Fig. 5). However, the C-shaped protofilaments formed by GSK3β-phosphorylated 4R tau in vitro adopt a more closed/compact shape as compared to the AD PHFs (Fig. 5*E*). In addition, the resolution of the fibril structure of GSK3β-phosphorylated tau is currently not sufficient to perform a more detailed comparison. Notably, a recent study reported that the tau PHF derived from the extracellular vesicles of an AD patient’s brain adopts a more compact fold as compared to the AD PHF isolated from total brain homogenates (*SI Appendix*, Fig. S12) (46). The compactness of the AD PHF isolated from the extracellular vesicles is due to the presence of an additional cofactor between two positively charged residues (R349 and K375). Consequently, the presence of different cofactors in different regions of the brain may drive tau to adopt an even more compact protofilament fold in certain subtypes of AD. Further work is also needed to understand the contribution of 3R tau isoforms to the formation of the AD tau strain.

Taken together, our study identifies specific C-terminal phosphorylation of tau as a major molecular factor leading to the formation of AD-like tau filament structure. It further strengthens the critical role played by the serine/threonine kinase GSK3β in the pathogenesis of AD.

## Materials and Methods

Detailed explanations regarding recombinant protein purification, phosphorylation of tau, aggregation assays, western blot analysis, protease digestion, microscopy, fluorescence recovery after photobleaching, NMR spectroscopy, in-gel digestion and extraction of peptides for mass spectrometry, negative-stain electron microscopy, and cryoelectron microscopy can be found in *SI Appendix*.

### Phosphorylation of Tau.

Phosphorylation of 200 µM tau was conducted using various kinases under specific conditions. Each reaction involved different buffers, ATP concentrations, and durations, with shaking at 30 or 37 °C. After phosphorylation, samples were boiled to precipitate the kinases, centrifuged, and the supernatant containing phosphorylated tau was dialyzed. Sequential phosphorylation was performed using the same protocol for each kinase.

### Aggregation Assays.

Unmodified and phosphorylated tau samples were aggregated using a cofactor-free protocol (29). Briefly, 25 µM of protein were aggregated at 37 °C in a specific buffer for 4 d in a 96-well plate with shaking and PTFE beads to promote fibrillization. Thioflavin-T (50 µM) was used to monitor aggregation kinetics.

### NMR Spectroscopy.

NMR experiments were conducted at 278 K to reduce amide-water proton exchange of tau (47). Various ^1^H-^15^N HSQC spectra of unmodified and phosphorylated tau were recorded using different spectrometers and conditions. Chemical shift assignments for 2N4R tau were previously reported (47), and cross-peaks of phosphorylated residues were identified through sequential assignment.

### Cryoelectron Microscopy.

For cryo-EM, 25 µM GSK3β-phosphorylated tau fibrils were sonicated, mixed with pronase, and quickly added to grids before plunge-freezing. Data were acquired using a Titan Krios G4 microscope, and images were processed for motion correction and CTF estimation. Manual fibril picking was done to train a model for automated picking. Fibrils were reconstructed using RELION-3.1.2 (34, 35), with several rounds of 3D classification and refinement to optimize helical parameters.

## Supplementary Material

Appendix 01 (PDF)

Dataset S01 (PDF)

## Data Availability

All study data are included in the article and/or supporting information.
