# Supplementary material for: GSK3β phosphorylation catalyzes the aggregation of tau into Alzheimer's disease-like filaments
Source: Proc Natl Acad Sci U S A. 2024 Dec 18;121(52):e2414176121. doi: 10.1073/pnas.2414176121 (PMC11670061; doi:10.1073/pnas.2414176121)
Supplement: Supplementary file 1 — Appendix 01 (PDF) [file pnas.2414176121.sapp.pdf]

Supplementary information for

GSK3β phosphorylation catalyzes the aggregation of Tau into Alzheimer's disease-like filaments

Pijush Chakraborty[1], Alain Ibáñez de Opakua[2], Jeffrey A. Purslow[2], Simon A. Fromm[3], Debdeep Chatterjee[2], Milan Zachrdla[2], Shannon Zhuang[2], Sambhavi Puri[4], Benjamin Wolozin[4,5,6], & Markus Zweckstetter[1,2,*]

[1] Department for NMR-based Structural Biology, Max Planck Institute for Multidisciplinary Sciences, Am Faßberg 11, 37077, Göttingen, Germany.
[2] German Center for Neurodegenerative Diseases (DZNE), Von-Siebold-Str. 3a, 37075 Göttingen, Germany.
[3] EMBL Imaging Centre, European Molecular Biology Laboratory, Meyerhofstr. 1, 69117, Heidelberg, Germany.
[4]Department of Pharmacology and Experimental Therapeutics, Boston University School of Medicine, Boston, MA, 02118

[5]Center for Neurophotonics, Boston University, Boston, MA 02215

[6]Center for Systems Neuroscience, Boston University, Boston, MA 02215

*Correspondence should be addressed to markus.zweckstetter@dzne.de

This PDF file includes:

Materials and methods

Figures S1 to S13

Table S1

Materials and methods

Protein purification

To prepare unlabeled 2N4R tau protein, a single colony from the LB-agar plate was taken and grown overnight in 50 mL LB medium supplemented with 100 μg/mL ampicillin at 37 °C. 22 mL of the overnight culture were transferred to 1L LB medium supplemented with 100 μg/mL ampicillin and allowed to grow until an OD_{600} of 0.8-0.9 was reached. Subsequently, the cells were induced with 0.5 mM IPTG and expressed for 1 hour.

To obtain uniformly ^{15}N-labeled 2N4R tau, cells were grown in 8 L LB until an OD_{600} of 0.6-0.8 was reached, then centrifuged at low speed (5,000 g), washed with 1X M9 salts, and resuspended in 2 L M9 minimal medium supplemented with 1 g/L $^{15}NH_4Cl$ as the only nitrogen source. After 1 hour, the cells were induced with 0.5 mM IPTG and expressed overnight at 37 ^{0}C.

After harvesting, cell pellets were resuspended in lysis buffer (20 mM MES (pH 6.8), 1 mM EGTA, 2 mM DTT) complemented with protease inhibitor mixture, 0.2 mM $MgCl_2$, lysozyme, and DNAse I. Subsequently, cells were disrupted with a French pressure cell press (in ice-cold conditions to avoid protein degradation). NaCl was added to a final concentration of 500 mM, and lysates were boiled for 20 minutes. Denatured proteins were removed by ultracentrifugation with 127,000 g at 4 °C for 30 minutes. To precipitate the DNA, 20 mg/mL streptomycin sulfate was added to the supernatant and incubated for 15 minutes at 4 °C followed by centrifugation at 15,000 g for 30 minutes. The pellet was discarded, and tau protein was precipitated by adding 0.361 g/mL ammonium sulfate to the supernatant, followed by centrifugation at 15,000 g for 30 minutes. The pellet containing tau protein was resuspended in buffer A (20 mM MES (pH 6.8), 1 mM EDTA, 2 mM DTT, 0.1 mM PMSF, 50 mM NaCl) and dialyzed against the same buffer (buffer A) to remove excess salt. The next day, the sample was filtered and applied to an equilibrated ion-exchange chromatography column (Mono S 10/100 GL, GE Healthcare), and weakly bound proteins were washed out with buffer A. Tau protein was eluted with a linear gradient of 60 % final concentration of buffer B (20 mM MES pH 6.8, 1 M NaCl, 1 mM EDTA, 2 mM DTT, 0.1 mM PMSF). Protein samples were concentrated by ultrafiltration (5 kDa Vivaspin, Sartorius) and further purified by reverse phase chromatography using a preparative C4 column (Vydac 214 TP, 5 μm, 8 x 250 mm) in an HPLC system coupled with ESI mass spectrometer. Protein purity was confirmed using mass spectrometry, and the purified protein was lyophilized and re-dissolved in the buffer of interest.

To prepare unlabeled GSK3β (kinase domain), a construct of human GSK3β (residues 35-386) with an N-terminal His-tag was cloned into a pET28a vector. A thrombin consensus sequence (LVPR/GS, where / signifies the cleaved peptide bond) was positioned between the His-tag and GSK3β components of the construct, leaving a four-residue overhang following the removal of the His-tag. The cloned vector was transformed into *E. coli* strain BL21-AI and subsequently plated onto an LB-agar plate treated with 50 µg/mL of Kanamycin for selection. A seed culture was produced by growing a single colony in 100 mL of Luria Broth (LB) medium overnight at 37 °C and 230 rpm. 20 mL of the seed culture was then inoculated into 1L of LB medium and grown at 37 °C and 110 rpm to an OD_{600} ~0.4, where the temperature was then reduced to 16 °C. At an OD_{600} ~0.6, recombinant protein expression was induced by the addition of 2 g/L (0.2%) of arabinose and 0.4 mM of Isopropyl ß-D-1-thiogalactopyranoside (IPTG). After 18 h of induction at 16 °C and 110 rpm, cells were harvested by centrifugation

for 30 min at 5,000 g. The supernatant was discarded, and the cell pellet resuspended in lysis buffer (50 mM HEPES (pH 7.2), 300 mM NaCl, 5% glycerol, 1 mM phenylmethylsulfonyl fluoride (PMSF), 20 µg/mL DNase, 0.1 mg/mL lysozyme, and EDTA-free protease inhibitor cocktail). The cell suspension was lysed on ice by sonication and subsequently centrifuged at 50,000 g for 30 min. The supernatant was decanted from cellular debris and passed through a 0.45 µM filter. The supernatant was loaded onto a 5 mL HisTrap HP prepacked column (Cytiva), washed with buffer A (50 mM HEPES (pH 7.2), 300 mM NaCl, 5% glycerol) and eluted using a gradient of buffer B (50 mM HEPES (pH 7.2), 300 mM NaCl, 5% glycerol, and 500 mM imidazole). Elution fractions were analyzed by SDS-polyacrylamide gel (SDS-PAGE) electrophoresis and all fractions containing target protein coalesced. The sample was then diluted with dilution buffer (50 mM HEPES (pH 7.2) and 5% glycerol) to reduce the salt concentration and loaded onto a Mono S 10/100 GL column (GE Healthcare). The column was washed with buffer C (50 mM HEPES (pH 7.2), 100 mM NaCl, and 5% glycerol), before eluting the target protein using a gradient of buffer D (50 mM HEPES (pH 7.2), 100 mM NaCl, 5% glycerol, and 1 M NaCl). The resulting elution was analyzed by SDS-PAGE electrophoresis, where the purist fractions were combined and concentrated using a 10K MWCO Vivaspin membrane filter (Sartorius). 72 units of thrombin per mg of protein were added and the sample was dialyzed overnight at 4 °C into 50 mM HEPES (pH 7.2), 150 mM NaCl, 5% glycerol, and 2 mM 2-Mercaptoethanol (BME). Following dialysis, the sample was loaded onto 1 mL of Ni-NTA beads equilibrated with buffer A to remove any uncleaved protein. The protein was then further purified using a HiLoad 16/600 Superdex 200 Size Exclusion Chromatography (SEC) column equilibrated with 20 mM MES (pH 6.5), 250 mM NaCl, 5% glycerol, and 2 mM BME. Following the complete purification, the target protein was calculated to be ~94% pure by analyzing an SDS-PAGE gel loaded with varying concentrations of protein.

Phosphorylation of tau

Phosphorylation reactions of tau in the presence of a single kinase were performed according to published protocols (MARK2cat – Schwalbe et al.(1), GSK3β and CaMKII – Ukmar-Godec et al.(2), PKA – Leroy et al.(3), ERK2 – Qi et al.(4), C-Abl – Savastano et al.(5)). The reaction time and the concentration of kinases were chosen such that the phosphorylation occurs predominantly on the sites that are phosphorylated most efficiently by the respective kinases (i.e., less phosphorylation on minor sites).

Phosphorylation of 200 µM tau was performed in the presence of 2.5 µM of MARK2cat WT kinase (purified in-house), 5 mM ATP, 1 mM Benzamidine, 2 mM EGTA, 1 mM PMSF in 25 mM Tris, 100 mM NaCl, 5 mM $MgCl_2$, 1 mM DTT, pH 8.0 buffer for a duration of 12 hours at 30 °C with 300 rpm shaking in an Eppendorf thermomixer.

Phosphorylation of 200 µM tau was performed with either 0.02 mg/ml of GSK3β (ab60863, Abcam) or 0.02 mg/ml of CDK5/p25 (C33-10G, SignalChem) and in the presence of 2 mM ATP, 5 mM EGTA, 1 mM PMSF in 40 mM HEPES, 5 mM $MgCl_2$, 2 mM DTT, pH 7.4 buffer for a duration of 12 hours at 30 °C with 300 rpm shaking in an Eppendorf thermomixer.

Phosphorylation of 200 µM tau was performed in the presence of 1 µM PKA (P6000S, NEB), 5 mM ATP, 5 mM EGTA, 1 mM PMSF in 50 mM HEPES, 12.5 mM $MgCl_2$, 50 mM

NaCl, 5 mM DTT, pH 8.0 buffer for a duration of 15 minutes at 30 °C with 300 rpm shaking in an Eppendorf thermomixer.

Phosphorylation of 200 µM tau was performed in the presence of 1 µM ERK2 (E1283, Sigma-Aldrich), 2.5 mM ATP, 2 mM EGTA, 1 mM PMSF in 50 mM HEPES, 12.5 mM $MgCl_2$, 50 mM NaCl, 2 mM DTT, pH 8.0 buffer for a duration of 3 hours at 37 °C with 300 rpm shaking in an Eppendorf thermomixer.

Phosphorylation of 200 µM tau was performed in the presence of 1 µM C-Abl (PR4348B, Life Technologies, Thermo Fischer Scientific), 5 mM ATP, 2 mM EGTA, 1 mM PMSF in 40 mM HEPES, 5 mM $MgCl_2$, 2 mM DTT, pH 7.4 buffer for a duration of 12 hours at 30 °C with 300 rpm shaking in an Eppendorf thermomixer.

Phosphorylation of 200 µM tau was performed in the presence of 0.02 mg/ml CaMKII (ab60899, Abcam), 2 mM ATP, 1 mM PMSF, 1 mM $CaCl_2$, 2 µM Calmodulin in 40 mM HEPES, 5 mM $MgCl_2$, 2 mM DTT, pH 7.4 buffer for a duration of 12 hours at 30 °C with 300 rpm shaking in an Eppendorf thermomixer.

At the end of the phosphorylation reactions, the samples were boiled at 98 °C for 20 minutes to precipitate the kinases followed by centrifugation at 20,000 g in an Eppendorf centrifuge 5424. Next, the pellet was discarded, and the supernatant containing phosphorylated tau was dialyzed against the buffer of interest.

The phosphorylation reactions with multiple kinases were performed sequentially, i.e., the phosphorylation reaction was performed with one kinase followed by the next one. In all cases, the same protocol mentioned for each kinase was used.

Aggregation assays

Unmodified as well as all phosphorylated tau samples were aggregated using the previously described co-factor free aggregation protocol(6). Briefly, 25 µM of protein were aggregated at 37 °C in 25 mM HEPES, 10 mM KCl, 5 mM $MgCl_2$, 3 mM TCEP, 0.01 % NaN_3, pH 7.2 buffer (aggregation assay buffer) in a 96 well plate using a Tecan spark plate reader for four days. Three PTFE beads along with double orbital shaking were used to promote fibrillization. Thioflavin-T (ThT) at a final concentration of 50 µM was used to monitor the aggregation kinetics.

Western blot analysis

50 µL of 25 µM GSK3β-phosphorylated/ unmodified tau fibrils were pelleted down by centrifugation at 20,000 g using an Eppendorf centrifuge 5424. The supernatant was discarded, and the pellet was redissolved in distilled water, separated by SDS-PAGE, and electro-transferred to nitrocellulose membranes using a Trans-Blot Turbo Transfer System (BIO-RAD) and subsequently blocked with 5 % BSA in phosphate buffer saline with 0.1 % Tween® 20 (PBST buffer) for 1 h and incubated with primary antibody (anti-phospho-tau (S396), ARG51604.50, Biomol) overnight at 4 °C. Next, the membrane was washed for 3 times with the PBST buffer and incubated with the secondary antibody (IRDye® 680RD Goat anti-Rabbit IgG, 926-68071, LI-COR) for 1 h at room temperature. The membrane was washed again three times with the PBST buffer and the immunoreactive bands were visualized by the LI-COR Odyssey CLx imaging system.

Protease digestion

50 µL of 0.8 mg/mL GSK3β-phosphorylated tau fibrils and 0.4 mg/ml of pronase (53702, Merck-Millipore), were incubated in the aggregation assay buffer for 30 minutes at 1,400 rpm shaking in an Eppendorf thermomixer at 37 °C. The pronase-resistant material was pelleted down by ultracentrifugation at 160,000 g for 30 minutes at 4 °C using a Beckman Coulter Optima MAX-UP ultracentrifuge. The supernatant was removed, and the pellet was redissolved in 10 µL of aggregation assay buffer and loaded in a 15 % SDS-PAGE gel. For mass spectrometry, the tau band from the SDS-PAGE gel was cut and digested by trypsin, followed by the detection of the peptides using an ESI mass spectrometer (Orbitrap Fusion Tribrid, Thermo Fischer Scientific).

Microscopy

For the experiments reported in Fig. 3b-g, 25 µM of unmodified or GSK3β/ERK2/CDK5/C-Abl phosphorylated tau in the aggregation assay buffer (25 mM HEPES, 10 mM KCl, 5 mM $MgCl_2$, 3 mM TCEP, 0.01 % NaN_3, pH 7.2) were incubated at room temperature for ten minutes and 5 µL of the sample was loaded onto a glass side and covered with an 18 mm coverslip for DIC microscopy experiments. For fluorescence microscopy experiments, the GSK3β-phosphorylated tau was labeled with Alexa-fluor-488 C_5 Maleimide (green) (Thermo Fischer Scientific) and 0.5 µL of the labeled protein were added to 15 µL of unlabeled protein, and from that mixture 5 µL of the sample were loaded onto a glass slide and covered with an 18 mm coverslip.

For the experiments reported in Fig. 4a, 25 µM tau were taken in 25 mM HEPES, 5 mM $MgCl_2$, pH 7.2 buffer in the presence of 10 % dextran and incubated at room temperature for 5 minutes. Then, Alexa-fluor-488 (Thermo Fischer Scientific) labeled GSK3β (Kinase domain) (in-house purified) was added to the solution at a final concentration of 0.02 mg/ml. 15 µL of the solution was then mixed with 0.5 µL of Alexa-fluor-594 (Thermo Fischer Scientific) labeled tau protein, and from that mixture 5 µL of the sample were loaded onto a glass slide and covered with an 18 mm coverslip.

For the experiments reported in Fig. 4b-d, 25 µM tau was taken in 25 mM HEPES, 5 mM $MgCl_2$, pH 7.2 buffer in the presence of 10 % dextran and incubated at room temperature for 5 minutes. Then, unlabeled GSK3β and ATP were added to the solution at a final concentration of 0.02 mg/ml and 1 mM, respectively and incubated at room temperature for measurement by microscopy at different time points. Before performing microscopy, 1 % of Alexa-fluor-594 (Thermo Fischer Scientific) labeled tau protein was added to the solution.

DIC and fluorescent micrographs were acquired on a Leica DM6B microscope with a 63x objective (water immersion) and processed using Fiji software (NIH).

Fluorescence recovery after photobleaching (FRAP)

FRAP experiments were recorded using a Zeiss LSM880 confocal microscope using a 63x objective (oil immersion) and either a 488 or 561 argon laser line. A circular region was chosen in a region of homogenous fluorescence and bleached with up to twelve iterations of full laser power and then the recovery was imaged. Pictures were analyzed in FIJI software (NIH) and FRAP recovery curves were calculated using standard methods based on fluorescence

intensities measured for pre-bleached/bleached, reference, and background ROI(7). The pre-bleached/bleached ROI was a selected region in the droplet before/after bleaching; the reference ROI correspond to an area that did not experience bleaching, while the background ROI correspond to an area where no fluorescence was detected. The intensity of the background ROI was further subtracted from the pre-bleached/bleached and reference ROIs. Briefly, the FRAP recovery was calculated as:

$$FRAP = \frac{\mathrm{Intensity(avg.\,prebleached)}}{Intensity(bleached) - Intensity(background)}$$

The value obtained was corrected by multiplying with the acquisition bleaching correction factor (ABCF) that was calculated according to

$$ABCF = \frac{\mathrm{Intensity(avg.\,prebleached)}}{Intensity(reference) - Intensity(background)}$$

The curves were then normalized according to the following equation:

$$Normalization = \frac{Intensity(t) - min.\,intensity\ value}{1 - \mathrm{min.\,intensity\ value}}$$

NMR Spectroscopy

All NMR experiments were recorded at 278K to reduce the amide-water proton exchange of tau(8).

^{1}H-^{15}N HSQC spectra of uniformly ^{15}N-labeled unmodified or GSK3β/CDK5/ERK2-phosphorylated tau (50 µM) were recorded in 50 mM NaP, 10 mM NaCl, 1 mM TCEP, pH 6.8 buffer at 278 K on an Avance III 900 MHz spectrometer (Bruker) using a 5 mm TCI (H/C/N) Cryoprobe. The spectra were collected with 40 scans per point (ns), and acquisition times td1 = 107.9 ms and td2 = 80.3 ms.

^{1}H-^{15}N HSQC spectra of uniformly ^{13}C-^{15}N-labeled unmodified K26 (30 µM) were recorded in 50 mM NaP, 10 mM NaCl, 1 mM TCEP, pH 6.8 buffer at 278 K on an Avance III 600 MHz spectrometer (Bruker) using a 5 mm QCI (H/C/N/F) Cryoprobe. The spectra were collected with 16 scans per point (ns), and acquisition times td1 = 43.8 ms and td2 = 155 ms.

^{1}H-^{15}N HSQC spectra of uniformly ^{13}C-^{15}N-labeled GSK3β-phosphorylated K26 (600 µM) were recorded in 50 mM NaP, 10 mM NaCl, 1 mM TCEP, pH 6.8 buffer at 278 K on an Avance III 600 MHz spectrometer (Bruker) using a 5 mm QCI (H/C/N/F) Cryoprobe. The spectra were collected with 8 scans per point (ns), and acquisition times td1 = 110.8 ms and td2 = 155 ms. The 3D HNCO spectra of the ^{13}C-^{15}N-labeled GSK3β-phosphorylated K26 was recorded on an Avance III 600 MHz spectrometer (Bruker) with 8 scans per point (ns), and acquisition times td1 = 47 ms , td2 = 22.5 ms, and td3 = 155.1 ms. A 3D HN(CA)CO spectrum was also recorded on the same spectrometer with 88 scans per point (ns), and acquisition times td1 = 23.5 ms, td2 = 20.7 ms, and td3 = 155.1 ms. A 3D HNCA spectrum of the ^{13}C-^{15}N-labeled GSK3β-phosphorylated K26 was recorded on an Avance Neo 800 MHz spectrometer (Bruker) using a 3 mm TCI (H/C/N) Cryoprobe with 16 scans per point (ns), and acquisition times td1

= 12.7 ms , td2 = 18.2 ms, and td3 = 118.8 ms. The 3D HN(CO)CA spectrum was recorded on the same spectrometer with 32 scans per point (ns), and acquisition times td1 = 12.7 ms, td2 = 12.9 ms, and td3 = 118.8 ms.

The chemical shift assignments of 2N4R tau had been previously reported(8). The spectra were recorded using Topsin 3.6.2/4.0.3 software (Bruker) and analyzed with CCPNMR 2.4.2 software (9). The cross-peaks of the phosphorylated S396, S400, and S404 were assigned by performing sequential assignment of the GSK3β-phosphorylated K26 protein. These assignments of the phosphorylated residues were transferred to the phosphorylated full-length (2N4R) tau.

Residue-specific intensity ratios were calculated according to intensity ratio = 1− (I/I_0), where I is the intensity of cross-peaks in the 2D ^{1}H-^{15}N HSQC spectrum of GSK3β/CDK5/ERK2-phosphorylated tau or GSK3β-phosphorylated K26 and I_0 is the intensity of the cross-peaks of unmodified tau or K26.

The chemical shift perturbation (CSP) was calculated according to:

$$CSP = \sqrt{0.5 * \{(\partial H)^2 + (\partial N/5)^2\}}$$

In-gel digestion and extraction of peptides for mass spectrometry

To determine the phosphorylation patterns of tau, the phosphorylated samples were loaded in an SDS-PAGE gel (Fig. S1). The respective bands from the SDS-PAGE gels were carefully cut and kept in an Eppendorf tube. To wash the gel pieces, 150 µL of water was added and incubated for 5 minutes at 26 °C with 1050 rpm shaking in a thermomixer. The gel pieces were spun down and the liquid was removed using thin tips (the same washing protocol was used in all subsequent steps with different solvents). The gel pieces were washed again with 150 µL acetonitrile. After washing, the gel pieces were dried for 5 minutes using a SpeedVacc vacuum centrifuge. To reduce disulfide bridges, 100 µL of 10 mM DTT was added to the gel pieces and incubated for 50 minutes at 56 °C followed by centrifugation and removal of liquid. The gel pieces were washed again with 150 µL of acetonitrile. To alkylate reduced cysteine residues, 100 µL of 55 mM iodoacetamide were added and incubated for 20 minutes at 26 °C with 1050 rpm shaking followed by centrifugation and removal of liquid. Subsequently, the gel pieces were washed with 150 µL of 100 mM NH_4HCO_3 , and then twice with 150 µL of acetonitrile and dried for 10 minutes in a vacuum centrifuge. The gel pieces were rehydrated at 4 °C for 45 minutes by addition of small amounts (2-5 µL) of digestion buffer 1 (12.5 µg/mL trypsin, 42 mM NH_4HCO_3, 4 mM $CaCl_2$). The samples were checked after every 15 minutes and more buffer was added in case the liquid was completely absorbed by the gel pieces. 20 µL of digestion buffer 2 (42 mM NH_4HCO_3, 4 mM $CaCl_2$) were added to cover the gel pieces and incubated overnight at 37 °C.

To extract the peptides, 15 µl water was added to the digest and incubated for 15 minutes at 37 °C with 1050 rpm shaking followed by spinning down the gel pieces. 50 µl acetonitrile was added to the entire mixture and incubated for 15 minutes at 37 °C with 1050 rpm shaking. The gel pieces were spun down and the supernatant (SN1) containing the extracted peptides was collected. 30 µl of 5 % (v/v) formic acid was added to the gel pieces and incubated for 15 minutes at 37 °C with 1050 rpm shaking followed by spinning down. Again 50 µl acetonitrile were added to the entire mixture and incubated for 15 minutes at 37 °C with 1050 rpm shaking. The gel pieces were spun down and the supernatant (SN2) containing the extracted peptides

was collected. Both supernatants (SN1 & SN2) containing the extracted peptides were pooled together and evaporated in the SpeedVacc vacuum centrifuge. The dried peptides were resuspended in 5 % acetonitrile and 0.1 % formic acid and analyzed using an Orbitrap Fusion Tribrid (Thermo Fischer Scientific) instrument. The MS data were analyzed using Scaffold 5.1.2 software. The protein threshold was set to 99 %, the peptide threshold was set to 95 %, and the minimum number of peptides was set to 1 which resulted in a peptide False Discovery Rate (FDR) of 0.36 %. The extent of phosphorylation for each residue was determined by the ratio between the number of detected peptides containing a particular phosphorylated residue and the total number of peptides detected containing the same residue (Dataset S1).

Negative-stain electron microscopy

40 µL of 25 µM unmodified or different phosphorylated tau fibrils were pelleted down by centrifugation at 20,000 g using an Eppendorf centrifuge 5424. The supernatant was discarded, and the pellet was redissolved in the aggregation assay buffer. Next, the fibrils were sonicated in a water bath (Bandelin sonorex) for 2 minutes. After sonication, 5.5 µL of fibril sample was mixed with 0.5 µL of 1 mg/mL pronase protease (53702, Merck-Millipore) followed by adsorbing onto carbon-coated copper grids. The samples were stained with 1% uranyl acetate solution and the images were taken with a Tietz F416 CMOS camera (TVIPS, Gauting, Germany) using a CM 120 transmission electron microscope (FEI, Eindhoven, The Netherlands).

Cryo-electron microscopy

For cryo-EM, 5 µL of 25 µM GSK3β-phosphorylated tau fibrils were sonicated for 1 minute using a water bath sonicator (Bandelin Sonorex digitec) and then mixed with 1 µL of 1 mg/mL pronase (53702, Merck-Millipore). The mixture was then quickly added to the Quantifoil 2/1 grids and plunged-frozen after an incubation of 5 seconds using a Leica EM GP2 automatic plunge freezer. Cryo-electron microscopy data were acquired with a Titan Krios G4 transmission-electron microscope (Thermo Fisher) operated at 300 keV accelerating voltage. Images were recorded using a Falcon 4i direct electron detector with a calibrated pixel size of 0.934 Å on the specimen level. The slit width of the Selectris X energy filter (Thermo Fisher) was set to 10 eV and a 100 µm objective aperture was inserted. In total, 9,009 images with defocus values in the range of -0.9 µm to -1.9 µm were acquired in movie mode with 2.7 s acquisition time. The accumulated dose was approximately 40 electrons per $Å^2$. The resulting dose-fractionated image stacks were subjected to beam-induced motion correction and CTF estimation.

Manual fibril picking was done with EMAN2 e2helixboxer(10) to select an average of ~5 segments per micrograph in 100 micrographs. The manual picking was used to train a model and pick the rest of the micrographs with crYOLO(11) with an inter-box distance of 19 Å.

GSK3β phosphorylated tau fibrils were reconstructed using RELION-3.1.2(12, 13), following the helical reconstruction scheme. For an initial 2D classification, we extracted particle segments using a box size of 1536 pixels downscaled to 192 pixels. Best classes with a visible twist were selected and used to estimate a crossover of around 1550 Å (Fig. 5b), which is equivalent to a twist of around -0.55° for a 4.75 Å rise. For 3D classification, the segments after 2D classification were re-extracted without downscaling using a box size of 400 pixels.

We performed several rounds of 3D classification starting from a 290 Å low-pass-filtered featureless cylinder and subsequent 3D refinements to optimize the helical parameters (rise of 4.77 Å and twist of -0.58°, reported in Table S1) and applying C2 symmetry. Next, standard RELION post-processing with a soft-edged solvent mask that includes the central 20 % of the box height yielded the final post-processed map (sharpening B-factor of -26.43 $Å^2$). The resolution (3.85 Å) was estimated from the value of the FSC curve for two independently refined half-maps at 0.143. The estimated resolution is overestimated because of the high resolution in the Z axis and the approximated real resolution is around 5 Å (See Fig. S11a,b). The 2D class average of the straight fibrils formed by GSK3β-phosphorylated tau is shown in Fig. S13.

Supplementary Figures

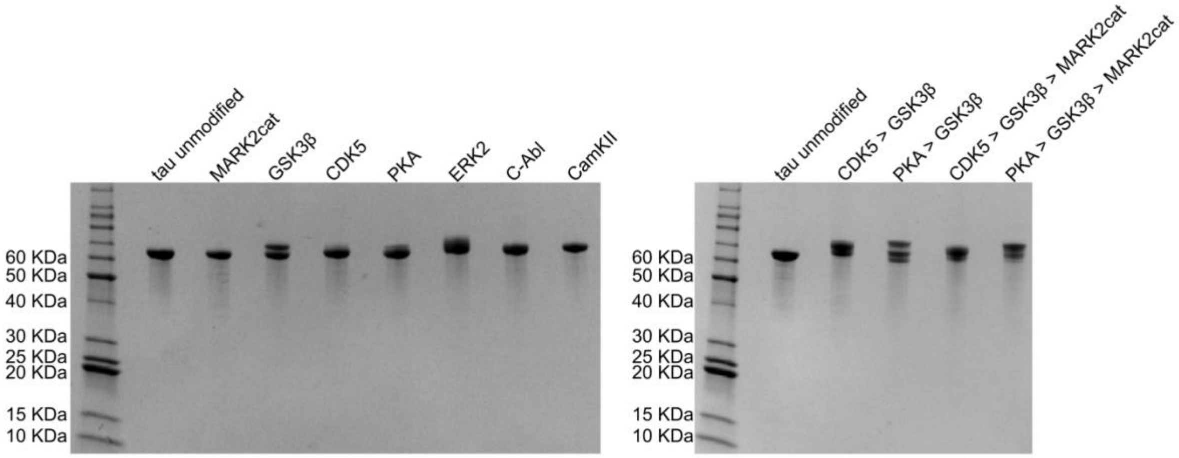

Fig. S1 │SDS-PAGE gel of unmodified and phosphorylated tau samples. The shift in the tau bands toward higher molecular weight indicates the phosphorylation of the sample. These bands are cut and digested by trypsin and the resulting peptides are analyzed by mass spectrometry. The phosphorylated residues identified by mass spectrometry are shown in Fig. 1a.

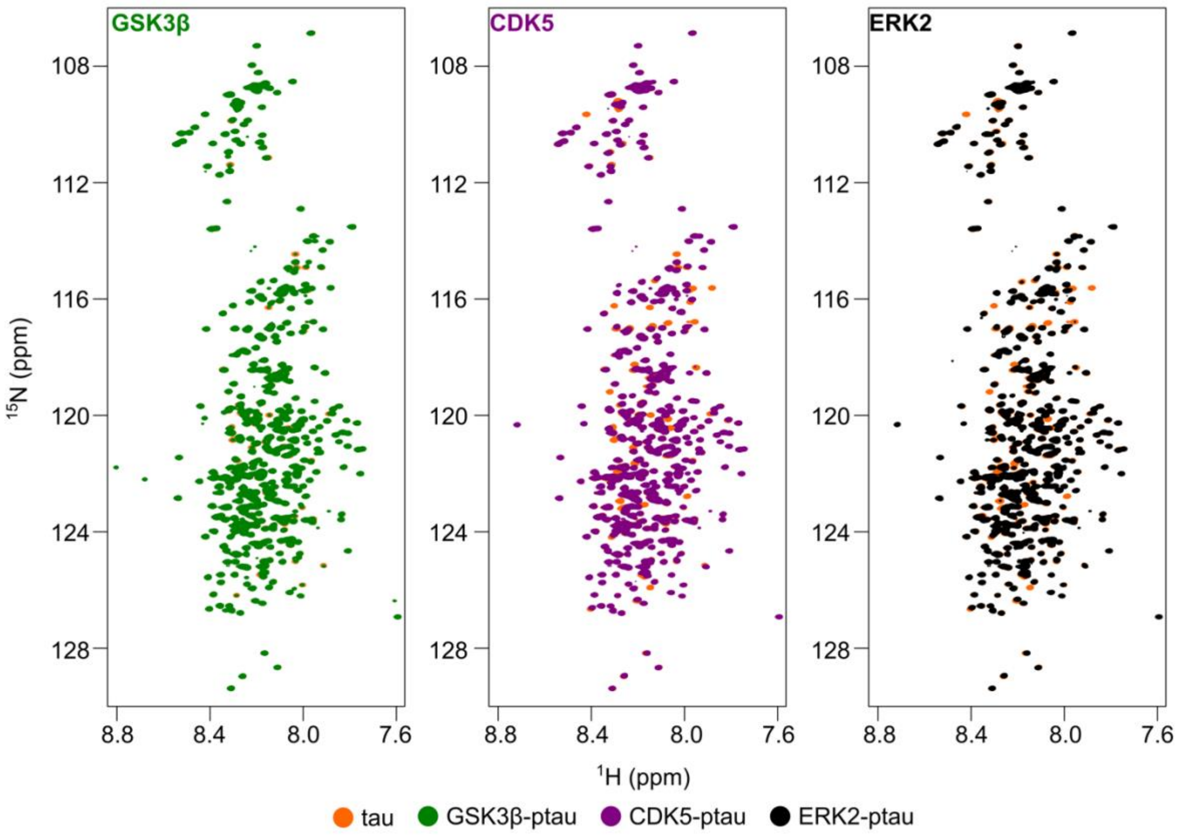

Fig. S2 │ ^{1}H-^{15}N HSQC spectra of unmodified and phosphorylated tau. The overlap of the HSQC spectra of unmodified tau (orange) and GSK3β (green), CDK5 (magenta), ERK2 (black)-phosphorylated tau is shown. A zoomed-in version of the HSQC spectra of phosphorylated tau is shown in Fig. 1e-g.

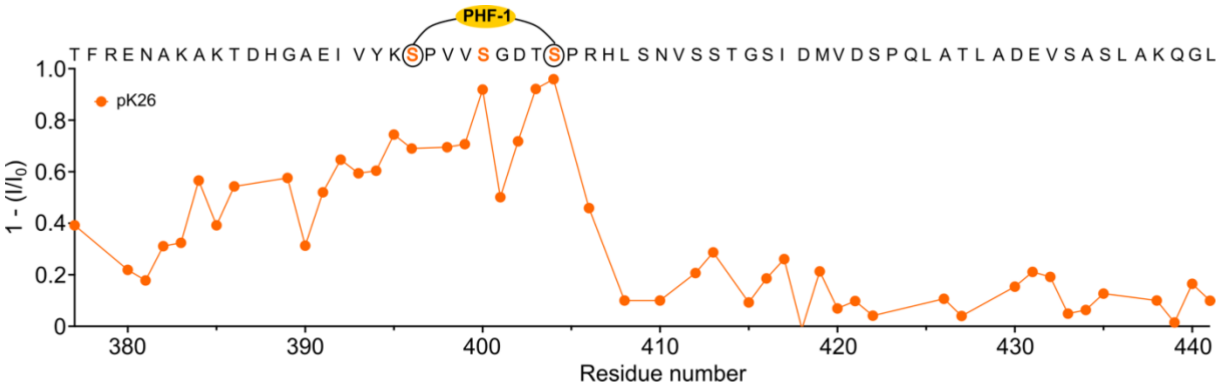

Fig. S3 | NMR spectroscopy of GSK3β-phosphorylated K26. Residue-specific intensity changes observed in the ^{1}H-^{15}N HSQC spectra (Fig. 1h) of K26 upon phosphorylation by GSK3β kinase.

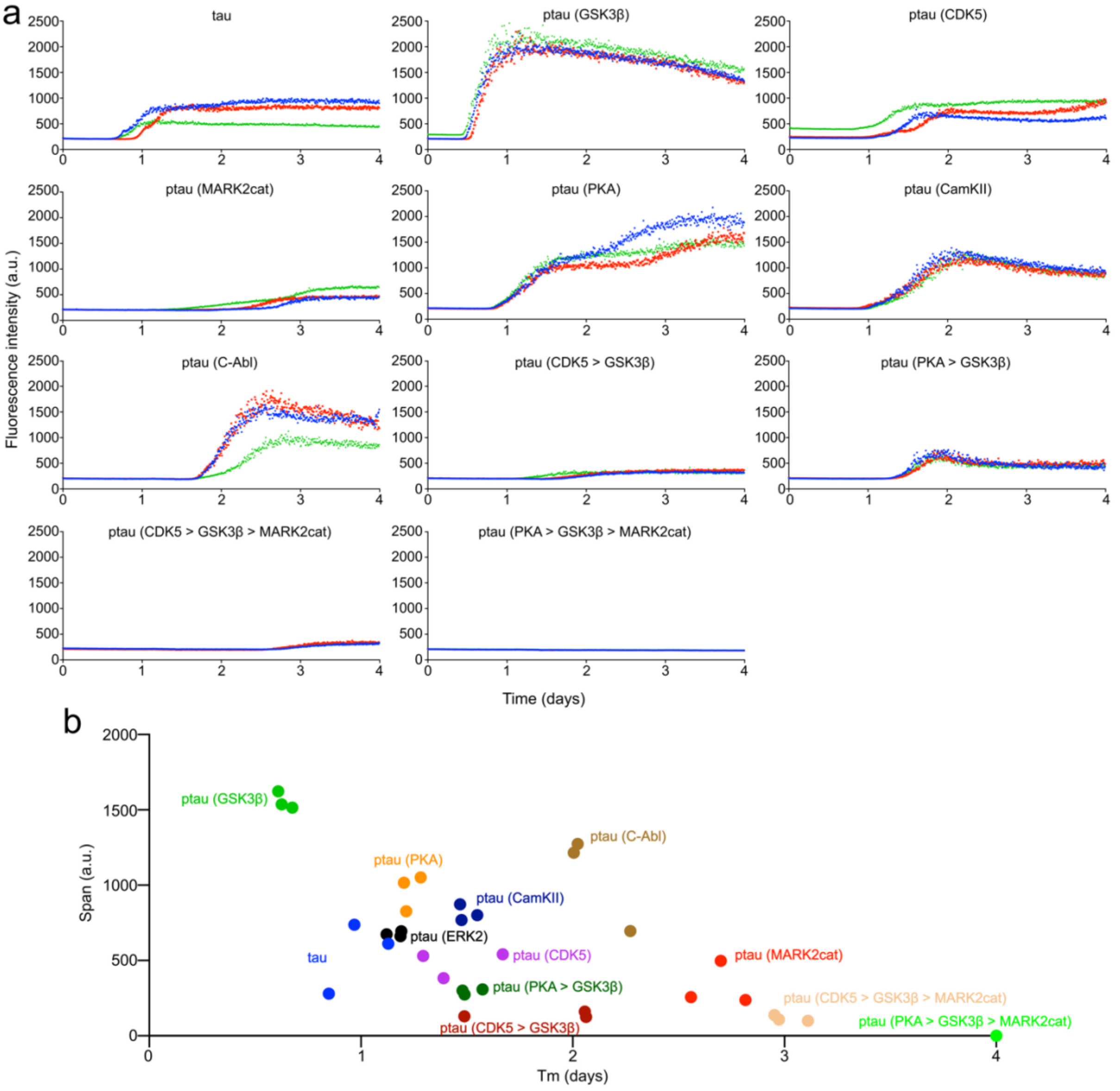

Fig. S4 │ Aggregation kinetics of different phosphorylated tau samples. a, Aggregation kinetics of three independent samples of 25 μM unmodified tau and tau phosphorylated by different kinases. **b,** ThT-intensity span vs. half time of aggregation (Tm) of unmodified and phosphorylated tau proteins.

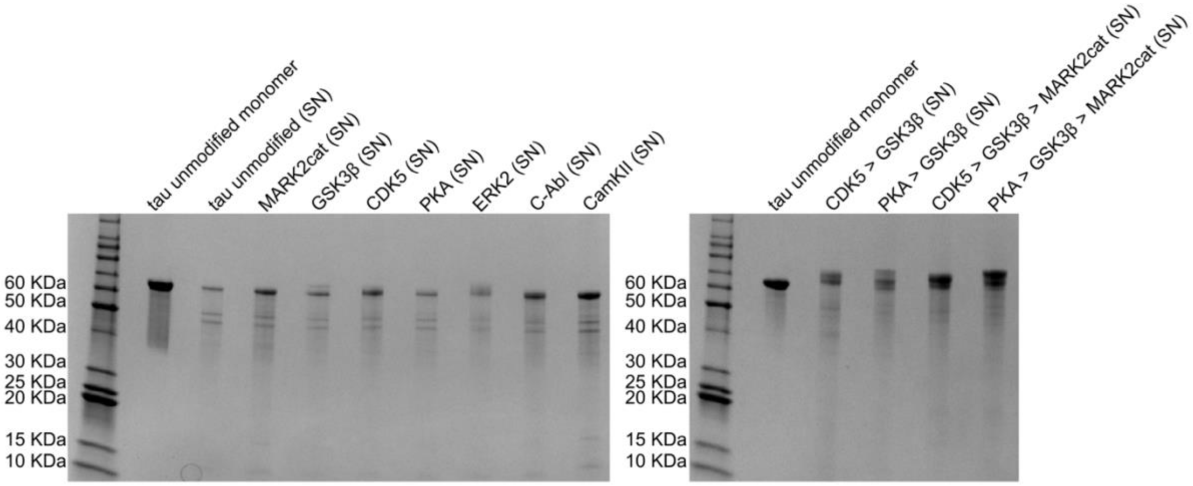

Fig. S5│ SDS-PAGE gel to determine the amount of aggregated protein. SDS-PAGE gel of unmodified tau monomer and supernatant (SN) (after pelleting down the fibrils) of unmodified and different phosphorylated tau samples. The fibril samples were collected after four days of aggregation. The amount of aggregated protein was calculated by comparing the intensity of the supernatant (SN) band to the tau monomer band.

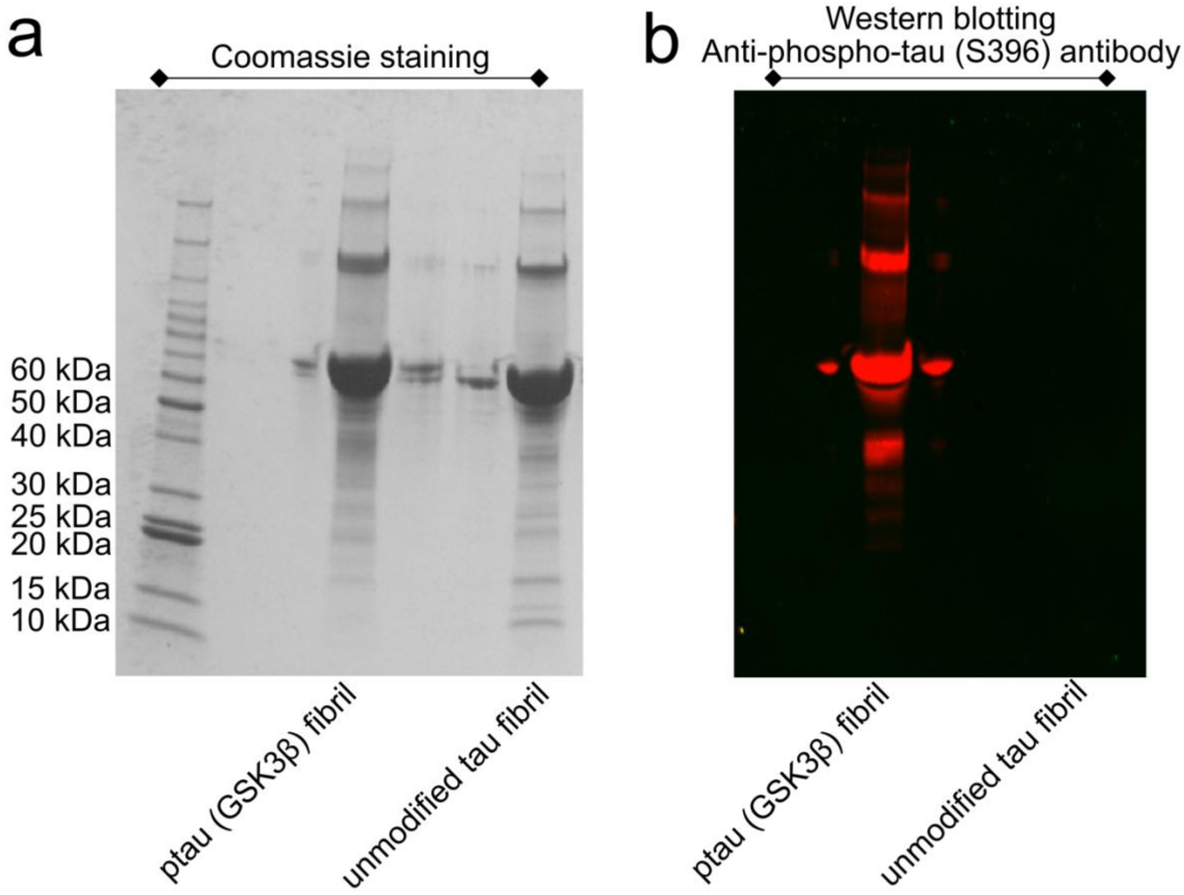

Fig. S6 │ Western blot analysis of GSK3β-ptau fibrils. a, SDS-PAGE gel of the GSK3β-ptau and unmodified tau fibrils. **b**, The anti-phospho-tau (S396) antibody selectively detected the fibrils formed by GSK3b-ptau and not the unmodified tau.

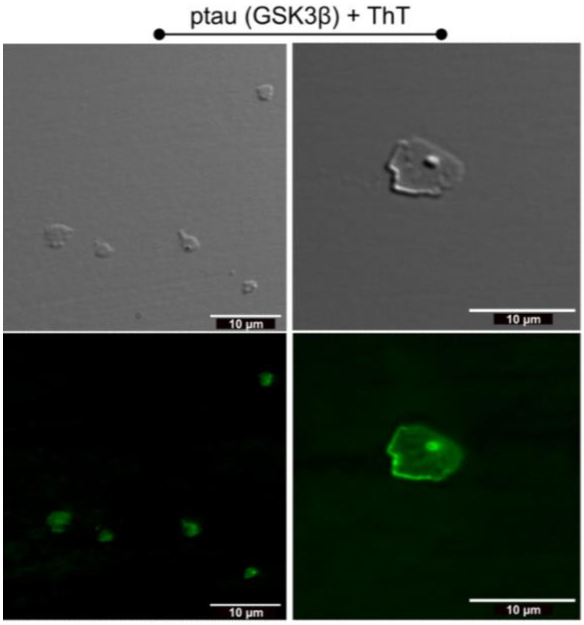

Fig. S7│ ThT staining of condensates formed by GSK3β-phosphorylated tau. DIC (top panel) and fluorescence microscopy (bottom panel) of condensates formed by GSK3β-phosphorylated tau. Scale bars, 10 μm.

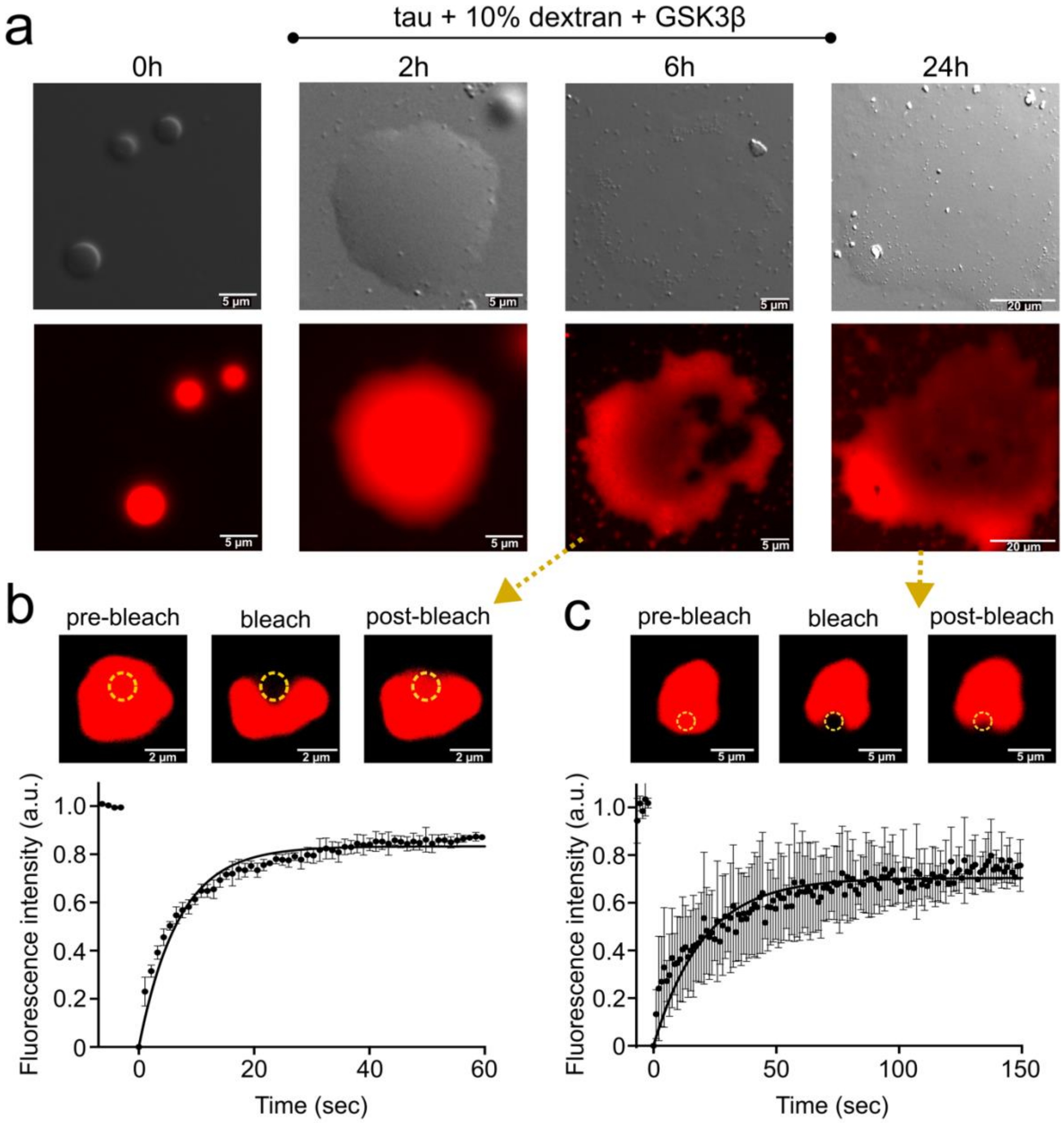

Fig. S8│ Incubation of tau droplets in the presence of GSK3β but without ATP. a, DIC and fluorescence microscopy of tau droplets induced by the addition of 10 % dextran at room temperature in 25 mM HEPES, 10 mM KCl, 5 mM $MgCl_2$, pH 7.2 buffer in the presence of 0.02 mg/ml unlabeled GSK3β (without ATP, i.e. under the conditions where no phosphorylation occurs). The sample was incubated for a duration of 24 hours. Micrographs are representative of three independent biological replicates. **b**, FRAP experiment of the tau condensates in the presence of GSK3β after incubation for six hours. The yellow arrow indicates that the FRAP experiments were performed on the condensates formed after incubation for six hours. Error bars represent the std of averaged three curves for each time point. Representative micrographs of the condensate before bleaching, after bleaching, and at the end of recovery are displayed (top panel). **c**, FRAP experiment of the tau condensates in the presence of GSK3β after incubation for twenty-four hours. The yellow arrow indicates that the FRAP experiments were performed on the condensates formed after incubation for twenty-four hours. Error bars represent the std of averaged three curves for each time point. Representative micrographs of the condensate before bleaching, after bleaching, and at the end of recovery are displayed (top panel).

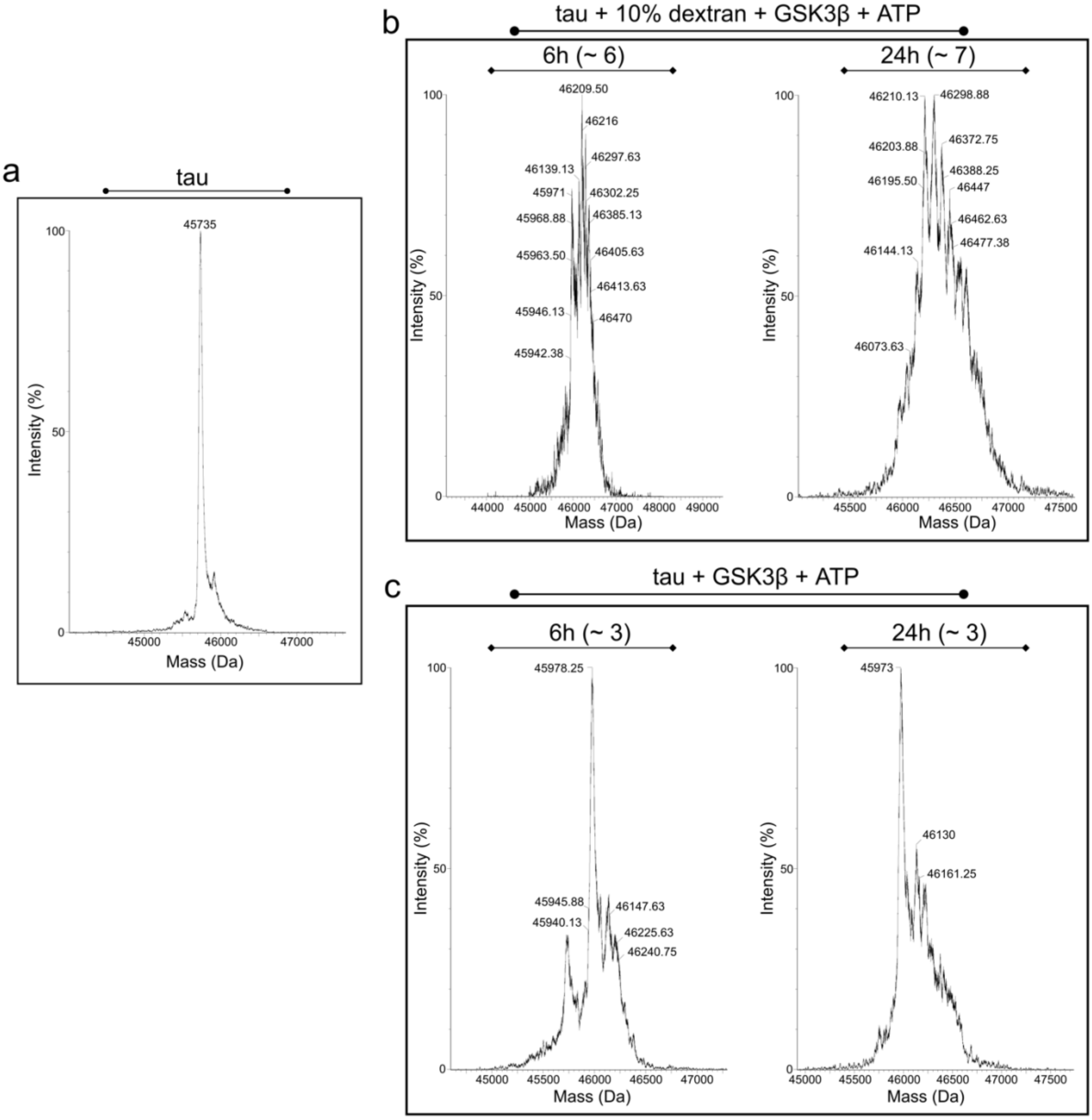

Fig. S9│ Mass spectrometry of tau phosphorylation. a, Mass spectra of monomeric tau protein. **b**, Mass spectra of tau droplets induced by the addition of 10 % dextran at room temperature in 25 mM HEPES, 5 mM $MgCl_2$, pH 7.2 buffer in the presence of 0.02 mg/ml unlabeled GSK3β and 1 mM ATP after incubation for six hours (left) and twenty-four hours (right). The average degree of phosphorylation is indicated within the brackets. **c**, Mass spectra of tau monomer at room temperature in 25 mM HEPES, 5 mM $MgCl_2$, pH 7.2 buffer in the presence of 0.02 mg/ml unlabeled GSK3β and 1 mM ATP after incubation for six hours (left) and twenty-four hours (right). The average degree of phosphorylation is indicated within the brackets.

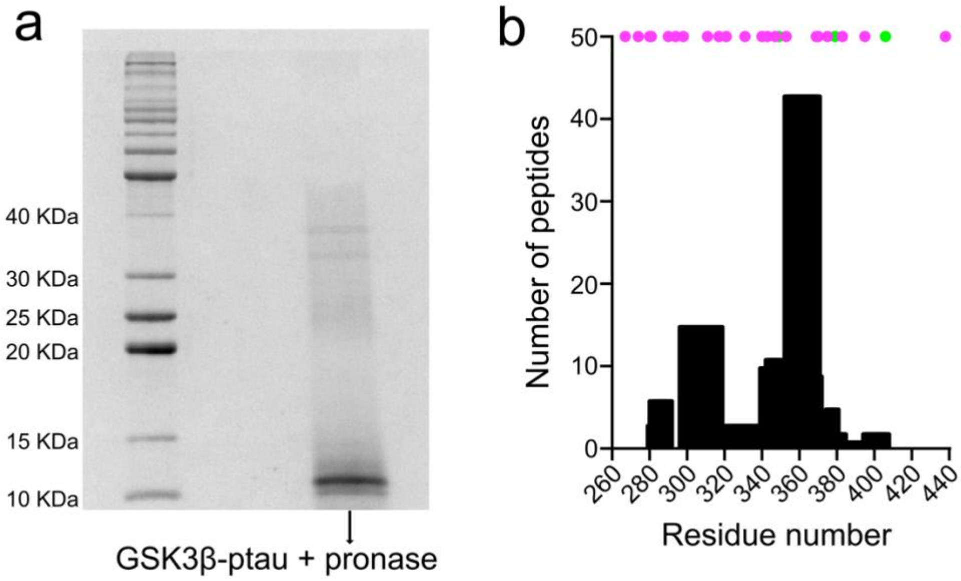

Fig. S10│ Determination of the location of the rigid core of GSK3β-phosphorylated tau fibrils. a, SDS-PAGE gel of pronase-digested GSK3β-phosphorylated tau fibrils. **b**, Numbers of peptides detected by mass spectrometry. The positions of lysine and arginine residues are marked with purple and green dots, respectively.

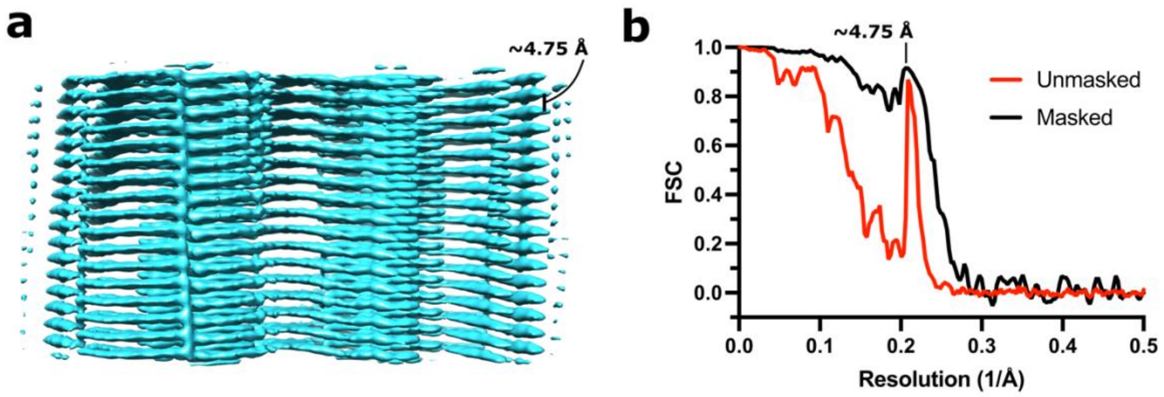

Fig. S11│ Cryo-EM of GSK3β-phosphorylated tau fibrils. a, Cryo-EM density map of GSK3β phosphorylated tau fibrils from the side view, showing the high z-axis resolution. **b,** Fourier shell correlation (FSC) curves of GSK3β phosphorylated tau fibril maps. FSC curves between two independently refined masked (black) and unmasked (red) half-maps. The final resolution estimated from the value of the FSC curve for two independently refined masked half-maps at 0.143 is 3.85 Å. The peak at 4.75 Å from the high z-axis resolution results in an overestimation of the resolution.

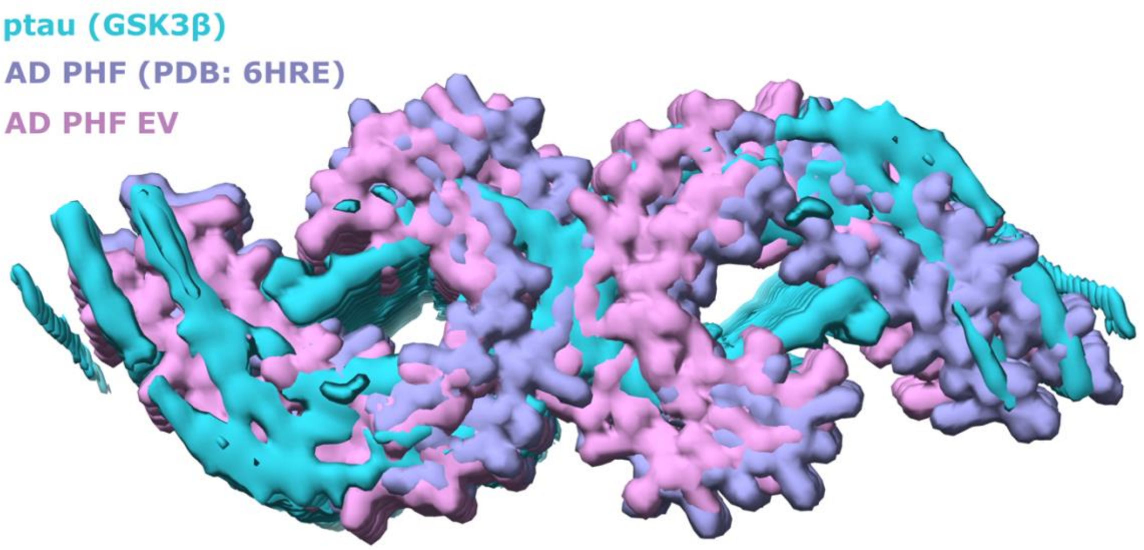

Fig. S12 │Superposition of the cryo-EM density maps of different tau fibrils. Cryo-EM density map of the GSK3β-phosphorylated tau fibrils (cyan surface) compared with the PHFs from sporadic Alzheimer's disease brain (dark blue surface; PDB id 6HRE) and the PHFs extracted from extracellular vesicles (EV) of Alzheimer's disease brain (pink surface) (ref #46).

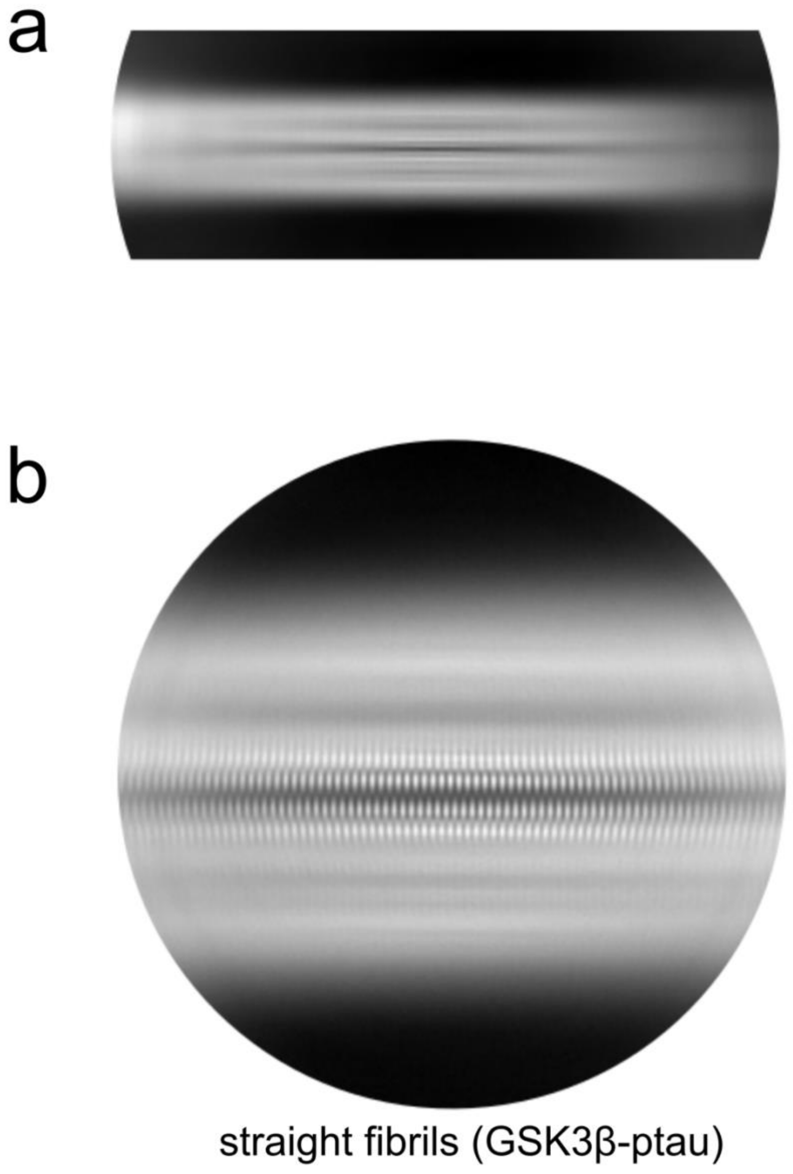

Fig. S13 │Cryo-EM of straight fibrils formed by GSK3β-phosphorylated tau. a, 2D class of the GSK3β-phosphorylated tau straight fibrils with big box and low resolution. **b**, 2D class of the GSK3β-phosphorylated tau straight fibrils with high resolution.

Supplementary Table

Table S1 | Cryo-EM collection and reconstruction data.

**Data collection**	
Microscope	Titan Krios G4
Voltage (kV)	300
Detector	Falcon 4i
Pixel size (Å)	0.934
Defocus range (µm)	-0.9 to -1.9
Exposure time (s)	2.7
Total dose (e^-/Å^2)	40
**Reconstruction**	
Picked segments	1,315,404
Box width (pixels)	400
Inter-box distance (pixels)	18
Final segments	21,444
Final resolution (Å)[a]	3.85 (~5)
Sharpening B-factor (Å^2)	-26.43
Symmetry imposed	C2
Helical rise (Å)	4.77
Helical twist (°)	-0.58

[a] The resolution was estimated from the value of the FSC curve for two independently refined half-maps at 0.143. The resolution is overestimated because of the high resolution in the Z-axis. In brackets is shown the approximated real resolution.

SI references

1. M. Schwalbe *et al.*, Phosphorylation of human Tau protein by microtubule affinity-regulating kinase 2. *Biochemistry* **52**, 9068-9079 (2013).
2. T. Ukmar-Godec *et al.*, Proteasomal degradation of the intrinsically disordered protein tau at single-residue resolution. *Sci Adv* **6**, eaba3916 (2020).
3. A. Leroy *et al.*, Spectroscopic studies of GSK3beta phosphorylation of the neuronal tau protein and its interaction with the N-terminal domain of apolipoprotein E. *J Biol Chem* **285**, 33435-33444 (2010).
4. H. Qi *et al.*, Characterization of Neuronal Tau Protein as a Target of Extracellular Signal-regulated Kinase. *J Biol Chem* **291**, 7742-7753 (2016).
5. A. Savastano *et al.*, Disease-Associated Tau Phosphorylation Hinders Tubulin Assembly within Tau Condensates. *Angew Chem Int Ed Engl* **60**, 726-730 (2021).
6. P. Chakraborty *et al.*, Co-factor-free aggregation of tau into seeding-competent RNA-sequestering amyloid fibrils. *Nat Commun* **12**, 4231 (2021).
7. A. Savastano, A. Ibanez de Opakua, M. Rankovic, M. Zweckstetter, Nucleocapsid protein of SARS-CoV-2 phase separates into RNA-rich polymerase-containing condensates. *Nat Commun* **11**, 6041 (2020).
8. M. D. Mukrasch *et al.*, Structural polymorphism of 441-residue tau at single residue resolution. *PLoS Biol* **7**, e34 (2009).
9. W. F. Vranken *et al.*, The CCPN data model for NMR spectroscopy: development of a software pipeline. *Proteins* **59**, 687-696 (2005).
10. G. Tang *et al.*, EMAN2: an extensible image processing suite for electron microscopy. *J Struct Biol* **157**, 38-46 (2007).
11. T. Wagner *et al.*, SPHIRE-crYOLO is a fast and accurate fully automated particle picker for cryo-EM. *Commun Biol* **2**, 218 (2019).
12. S. He, S. H. W. Scheres, Helical reconstruction in RELION. *J Struct Biol* **198**, 163-176 (2017).
13. S. H. W. Scheres, Amyloid structure determination in RELION-3.1. *Acta Crystallogr D Struct Biol* **76**, 94-101 (2020).
