# Supplementary material for: GSK3β phosphorylation catalyzes the aggregation of tau into Alzheimer's disease-like filaments
Source: Proc Natl Acad Sci U S A. 2024 Dec 18;121(52):e2414176121. doi: 10.1073/pnas.2414176121 (PMC11670061; doi:10.1073/pnas.2414176121)
Supplement: Supplementary file 2 — Dataset S01 (PDF) [file pnas.2414176121.sd01.pdf]

Supplementary Data

Dataset S1 │Determination of residue-specific phosphorylation level of tau by Mass Spectrometry. The peptide coverage is shown by highlighting the residues with yellow colour on the sequence of full-length tau. The phosphorylated residues are highlighted with green colour. The extent of phosphorylation for each residue was determined by the ratio between the number of detected peptides containing a particular phosphorylated residue and the total number of peptides detected containing the same residue.

htau40 (GSK3β)

```
        10         20         30         40         50         60
MAEPRQEFEV MEDHAGTYGL GDRKDQGGYT MHQDQEGDTD AGLKESPLQT PTEDGSEEPG

        70         80         90        100        110        120
SETSDAKSTP TAEDVTAPLV DEGAPGKQAA AQPHTEIPEG TTAEEAGIGD TPSLEDEAAG

       130        140        150        160        170        180
HVTQARMVSK SKDGTGSDDK KAKGADGKTK IATPRGAAPP GQKGQANATR IPAKTPPAPK

       190        200        210        220        230        240
TPPSSGEPPK SGDRSGYSSP GSPGTPGSRS RTPSLPTPPT REPKKVAVVR TPPKSPSSAK

       250        260        270        280        290        300
SRLQTAPVPM PDLKNVKSKI GSTENLKHQP GGGKVQIINK KLDLSNVQSK CGSKDNIKHV

       310        320        330        340        350        360
PGGGSVQIVY KPVDLSKVTS KCGSLGNIHH KPGGGQVEVK SEKLDFKDRV QSKIGSLDNI

       370        380        390        400        410        420
THVPGGGNKK IETHKLTFRE NAKAKTDHGA EIVYKSPVVS GDTSPRHLSN VSSTGSIDMV

       430        440
DSPQLATLAD EVSASLAKQG L
```

Residue	Total number of peptides detected containing a particular residue	Number of peptides detected containing a particular phosphorylated residue	% of phosphorylation
T175	6	1	16.67
T181	2	1	50.00
T205	8	1	12.50
T231	4	1	25.00
S404	10	3	30.00

htau40 (CDK5)

```
        10         20         30         40         50         60
MAEPRQEFEV MEDHAGTYGL GDRKDQGGYT MHQDQEGDTD AGLKESPLQT PTEDGSEEPG

        70         80         90        100        110        120
SETSDAKSTP TAEDVTAPLV DEGAPGKQAA AQPHTEIPEG TTAEEAGIGD TPSLEDEAAG

       130        140        150        160        170        180
HVTQARMVSK SKDGTGSDDK KAKGADGKTK IATPRGAAPP GQKGQANATR IPAKTPPAPK

       190        200        210        220        230        240
TPPSSGEPPK SGDRSGYSSP GSPGTPGSRS RTPSLPTPPT REPKKVAVVR TPPKSPSSAK

       250        260        270        280        290        300
SRLQTAPVPM PDLKNVKSKI GSTENLKHQP GGGKVQIINK KLDLSNVQSK CGSKDNIKHV

       310        320        330        340        350        360
PGGGSVQIVY KPVDLSKVTS KCGSLGNIHH KPGGGQVEVK SEKLDFKDRV QSKIGSLDNI

       370        380        390        400        410        420
THVPGGGNKK IETHKLTFRE NAKAKTDHGA EIVYKSPVVS GDTSPRHLSN VSSTGSIDMV

       430        440
DSPQLATLAD EVSASLAKQG L
```

Residue	Total number of peptides detected containing a particular residue	Number of peptides detected containing a particular phosphorylated residue	% of phosphorylation
T175	7	1	14.29
T181	4	1	25.00
S202	6	2	33.33
T205	6	2	33.33
T212	33	6	18.18
S214	33	1	3.03
T217	33	2	6.06
T220	33	1	3.03
T231	2	1	50.00
S235	2	2	100.00
S404	20	9	45.00

htau40 (ERK2)

```
        10         20         30         40         50         60
MAEPRQEFEV MEDHAGTYGL GDRKDQGGYT MHQDQEGDTD AGLKESPLQT PTEDGSEEPG

        70         80         90        100        110        120
SETSDAKSTP TAEDVTAPLV DEGAPGKQAA AQPHTEIPEG TTAEEAGIGD TPSLEDEAAG

       130        140        150        160        170        180
HVTQARMVSK SKDGTGSDDK KAKGADGKTK IATPRGAAPP GQKGQANATR IPAKTPPAPK

       190        200        210        220        230        240
TPPSSGEPPK SGDRSGYSSP GSPGTPGSRS RTPSLPTPPT REPKKVAVVR TPPKSPSSAK

       250        260        270        280        290        300
SRLQTAPVPM PDLKNVKSKI GSTENLKHQP GGGKVQIINK KLDLSNVQSK CGSKDNIKHV

       310        320        330        340        350        360
PGGGSVQIVY KPVDLSKVTS KCGSLGNIHH KPGGGQVEVK SEKLDFKDRV QSKIGSLDNI

       370        380        390        400        410        420
THVPGGGNKK IETHKLTFRE NAKAKTDHGA EIVYKSPVVS GDTSPRHLSN VSSTGSIDMV

       430        440
DSPQLATLAD EVSASLAKQG L
```

Residue	Total number of peptides detected containing a particular residue	Number of peptides detected containing a particular phosphorylated residue	% of phosphorylation
T175	18	5	27.78
T181	15	11	73.33
S202	7	1	14.29
T205	7	3	42.86
S210	13	2	15.38
T212	29	5	17.24
S214	29	2	6.90
T217	29	1	3.45
S235	1	1	100.00
T245	18	1	5.56
S396	19	1	5.26
T403	19	1	5.26
S404	19	7	36.84

htau40 (MARK2cat)

```
        10         20         30         40         50         60
MAEPRQEFEV MEDHAGTYGL GDRKDQGGYT MHQDQEGDTD AGLKESPLQT PTEDGSEEPG

        70         80         90        100        110        120
SETSDAKSTP TAEDVTAPLV DEGAPGKQAA AQPHTEIPEG TTAEEAGIGD TPSLEDEAAG

       130        140        150        160        170        180
HVTQARMVSK SKDGTGSDDK KAKGADGKTK IATPRGAAPP GQKGQANATR IPAKTPPAPK

       190        200        210        220        230        240
TPPSSGEPPK SGDRSGYSSP GSPGTPGSRS RTPSLPTPPT REPKKVAVVR TPPKSPSSAK

       250        260        270        280        290        300
SRLQTAPVPM PDLKNVKSKI GSTENLKHQP GGGKVQIINK KLDLSNVQSK CGSKDNIKHV

       310        320        330        340        350        360
PGGGSVQIVY KPVDLSKVTS KCGSLGNIHH KPGGGQVEVK SEKLDFKDRV QSKIGSLDNI

       370        380        390        400        410        420
THVPGGGNKK IETHKLTFRE NAKAKTDHGA EIVYKSPVVS GDTSPRHLSN VSSTGSIDMV

       430        440
DSPQLATLAD EVSASLAKQG L
```

Residue	Total number of peptides detected containing a particular residue	Number of peptides detected containing a particular phosphorylated residue	% of phosphorylation
S262	9	3	33.33
S356	11	1	9.09

htau40 (PKA)

```
        10         20         30         40         50         60
MAEPRQEFEV MEDHAGTYGL GDRKDQGGYT MHQDQEGDTD AGLKESPLQT PTEDGSEEPG

        70         80         90        100        110        120
SETSDAKSTP TAEDVTAPLV DEGAPGKQAA AQPHTEIPEG TTAEEAGIGD TPSLEDEAAG

       130        140        150        160        170        180
HVTQARMVSK SKDGTGSDDK KAKGADGKTK IATPRGAAPP GQKGQANATR IPAKTPPAPK

       190        200        210        220        230        240
TPPSSGEPPK SGDRSGYSSP GSPGTPGSRS RTPSLPTPPT REPKKVAVVR TPPKSPSSAK

       250        260        270        280        290        300
SRLQTAPVPM PDLKNVKSKI GSTENLKHQP GGGKVQIINK KLDLSNVQSK CGSKDNIKHV

       310        320        330        340        350        360
PGGGSVQIVY KPVDLSKVTS KCGSLGNIHH KPGGGQVEVK SEKLDFKDRV QSKIGSLDNI

       370        380        390        400        410        420
THVPGGGNKK IETHKLTFRE NAKAKTDHGA EIVYKSPVVS GDTSPRHLSN VSSTGSIDMV

       430        440
DSPQLATLAD EVSASLAKQG L
```

Residue	Total number of peptides detected containing a particular residue	Number of peptides detected containing a particular phosphorylated residue	% of phosphorylation
T212	31	2	6.45
S214	31	18	58.06
T217	31	1	3.23
S305	13	1	7.69
S356	20	1	5.00

htau40 (CamKII)

```
        10         20         30         40         50         60
MAEPRQEFEV MEDHAGTYGL GDRKDQGGYT MHQDQEGDTD AGLKESPLQT PTEDGSEEPG

        70         80         90        100        110        120
SETSDAKSTP TAEDVTAPLV DEGAPGKQAA AQPHTEIPEG TTAEEAGIGD TPSLEDEAAG

       130        140        150        160        170        180
HVTQARMVSK SKDGTGSDDK KAKGADGKTK IATPRGAAPP GQKGQANATR IPAKTPPAPK

       190        200        210        220        230        240
TPPSSGEPPK SGDRSGYSSP GSPGTPGSRS RTPSLPTPPT REPKKVAVVR TPPKSPSSAK

       250        260        270        280        290        300
SRLQTAPVPM PDLKNVKSKI GSTENLKHQP GGGKVQIINK KLDLSNVQSK CGSKDNIKHV

       310        320        330        340        350        360
PGGGSVQIVY KPVDLSKVTS KCGSLGNIHH KPGGGQVEVK SEKLDFKDRV QSKIGSLDNI

       370        380        390        400        410        420
THVPGGGNKK IETHKLTFRE NAKAKTDHGA EIVYKSPVVS GDTSPRHLSN VSSTGSIDMV

       430        440
DSPQLATLAD EVSASLAKQG L
```

Residue	Total number of peptides detected containing a particular residue	Number of peptides detected containing a particular phosphorylated residue	% of phosphorylation
S214	24	4	16.67
T263	10	1	10.00
Y310	9	1	11.11
S356	16	2	12.50
Y396	16	2	12.50

htau40 (C-Abl)

```
        10         20         30         40         50         60
MAEPRQEFEV MEDHAGTYGL GDRKDQGGYT MHQDQEGDTD AGLKESPLQT PTEDGSEEPG

        70         80         90        100        110        120
SETSDAKSTP TAEDVTAPLV DEGAPGKQAA AQPHTEIPEG TTAEEAGIGD TPSLEDEAAG

       130        140        150        160        170        180
HVTQARMVSK SKDGTGSDDK KAKGADGKTK IATPRGAAPP GQKGQANATR IPAKTPPAPK

       190        200        210        220        230        240
TPPSSGEPPK SGDRSGYSSP GSPGTPGSRS RTPSLPTPPT REPKKVAVVR TPPKSPSSAK

       250        260        270        280        290        300
SRLQTAPVPM PDLKNVKSKI GSTENLKHQP GGGKVQIINK KLDLSNVQSK CGSKDNIKHV

       310        320        330        340        350        360
PGGGSVQIVY KPVDLSKVTS KCGSLGNIHH KPGGGQVEVK SEKLDFKDRV QSKIGSLDNI

       370        380        390        400        410        420
THVPGGGNKK IETHKLTFRE NAKAKTDHGA EIVYKSPVVS GDTSPRHLSN VSSTGSIDMV

       430        440
DSPQLATLAD EVSASLAKQG L
```

Residue	Total number of peptides detected containing a particular residue	Number of peptides detected containing a particular phosphorylated residue	% of phosphorylation
S195	10	4	40.00
Y197	10	1	10.00
T263	7	1	14.29
Y310	11	2	18.18
Y394	14	6	42.86
S396	15	1	6.67

htau40 (Cdk5 -> GSK3b)

```
        10         20         30         40         50         60
MAEPRQEFEV MEDHAGTYGL GDRKDQGGYT MHQDQEGDTD AGLKESPLQT PTEDGSEEPG

        70         80         90        100        110        120
SETSDAKSTP TAEDVTAPLV DEGAPGKQAA AQPHTEIPEG TTAEEAGIGD TPSLEDEAAG

       130        140        150        160        170        180
HVTQARMVSK SKDGTGSDDK KAKGADGKTK IATPRGAAPP GQKGQANATR IPAKTPPAPK

       190        200        210        220        230        240
TPPSSGEPPK SGDRSGYSSP GSPGTPGSRS RTPSLPTPPT REPKKVAVVR TPPKSPSSAK

       250        260        270        280        290        300
SRLQTAPVPM PDLKNVKSKI GSTENLKHQP GGGKVQIINK KLDLSNVQSK CGSKDNIKHV

       310        320        330        340        350        360
PGGGSVQIVY KPVDLSKVTS KCGSLGNIHH KPGGGQVEVK SEKLDFKDRV QSKIGSLDNI

       370        380        390        400        410        420
THVPGGGNKK IETHKLTFRE NAKAKTDHGA EIVYKSPVVS GDTSPRHLSN VSSTGSIDMV

       430        440
DSPQLATLAD EVSASLAKQG L
```

Residue	Total number of peptides detected containing a particular residue	Number of peptides detected containing a particular phosphorylated residue	% of phosphorylation
T175	12	4	33.33
T181	7	5	71.43
S198	7	1	14.29
S202	7	6	85.71
T205	7	3	42.86
T212	92	3	3.26
S214	92	3	3.26
T217	92	5	5.43
T220	92	1	1.09
S305	16	1	6.25
S324	1	1	100.00
S400	5	1	20.00
S404	5	2	40.00

htau40 (PKA -> GSK3b)

```
        10         20         30         40         50         60
MAEPRQEFEV MEDHAGTYGL GDRKDQGGYT MHQDQEGDTD AGLKESPLQT PTEDGSEEPG

        70         80         90        100        110        120
SETSDAKSTP TAEDVTAPLV DEGAPGKQAA AQPHTEIPEG TTAEEAGIGD TPSLEDEAAG

       130        140        150        160        170        180
HVTQARMVSK SKDGTGSDDK KAKGADGKTK IATPRGAAPP GQKGQANATR IPAKTPPAPK

       190        200        210        220        230        240
TPPSSGEPPK SGDRSGYSSP GSPGTPGSRS RTPSLPTPPT REPKKVAVVR TPPKSPSSAK

       250        260        270        280        290        300
SRLQTAPVPM PDLKNVKSKI GSTENLKHQP GGGKVQIINK KLDLSNVQSK CGSKDNIKHV

       310        320        330        340        350        360
PGGGSVQIVY KPVDLSKVTS KCGSLGNIHH KPGGGQVEVK SEKLDFKDRV QSKIGSLDNI

       370        380        390        400        410        420
THVPGGGNKK IETHKLTFRE NAKAKTDHGA EIVYKSPVVS GDTSPRHLSN VSSTGSIDMV

       430        440
DSPQLATLAD EVSASLAKQG L
```

Residue	Total number of peptides detected containing a particular residue	Number of peptides detected containing a particular phosphorylated residue	% of phosphorylation
T175	7	3	42.86
T181	3	1	33.33
S199	16	1	6.25
S210	7	4	57.14
S214	58	24	41.38
T231	2	1	50.00
S305	16	1	6.25
S324	3	3	100.00
S356	30	1	3.33
S396	12	2	16.67
S400	12	1	8.33
T403	12	1	8.33

htau40 (Cdk5 -> GSK3b -> MARK2cat)

```
        10         20         30         40         50         60
MAEPRQEFEV MEDHAGTYGL GDRKDQGGYT MHQDQEGDTD AGLKESPLQT PTEDGSEEPG

        70         80         90        100        110        120
SETSDAKSTP TAEDVTAPLV DEGAPGKQAA AQPHTEIPEG TTAEEAGIGD TPSLEDEAAG

       130        140        150        160        170        180
HVTQARMVSK SKDGTGSDDK KAKGADGKTK IATPRGAAPP GQKGQANATR IPAKTPPAPK

       190        200        210        220        230        240
TPPSSGEPPK SGDRSGYSSP GSPGTPGSRS RTPSLPTPPT REPKKVAVVR TPPKSPSSAK

       250        260        270        280        290        300
SRLQTAPVPM PDLKNVKSKI GSTENLKHQP GGGKVQIINK KLDLSNVQSK CGSKDNIKHV

       310        320        330        340        350        360
PGGGSVQIVY KPVDLSKVTS KCGSLGNIHH KPGGGQVEVK SEKLDFKDRV QSKIGSLDNI

       370        380        390        400        410        420
THVPGGGNKK IETHKLTFRE NAKAKTDHGA EIVYKSPVVS GDTSPRHLSN VSSTGSIDMV

       430        440
DSPQLATLAD EVSASLAKQG L
```

Residue	Total number of peptides detected containing a particular residue	Number of peptides detected containing a particular phosphorylated residue	% of phosphorylation
T71	22	1	4.55
T181	7	4	57.14
S199	6	1	16.67
S202	6	4	66.67
T205	6	2	33.33
S208	6	1	16.67
T212	75	3	4.00
S214	75	2	2.67
T217	75	5	6.67
S262	7	1	14.29
T263	7	2	28.57
S305	13	1	7.69
S324	2	2	100.00
S356	24	5	20.83
S396	5	3	60.00
S400	5	1	20.00
S404	5	1	20.00

htau40 (PKA -> GSK3b -> MARK2cat)

```
         10         20         30         40         50         60
MAEPRQEFEV MEDHAGTYGL GDRKDQGGYT MHQDQEGDTD AGLKESPLQT PTEDGSEEPG

         70         80         90        100        110        120
SETSDAKSTP TAEDVTAPLV DEGAPGKQAA AQPHTEIPEG TTAEEAGIGD TPSLEDEAAG

        130        140        150        160        170        180
HVTQARMVSK SKDGTGSDDK KAKGADGKTK IATPRGAAPP GQKGQANATR IPAKTPPAPK

        190        200        210        220        230        240
TPPSSGEPPK SGDRSGYSSP GSPGTPGSRS RTPSLPTPPT REPKKVAVVR TPPKSPSSAK

        250        260        270        280        290        300
SRLQTAPVPM PDLKNVKSKI GSTENLKHQP GGGKVQIINK KLDLSNVQSK CGSKDNIKHV

        310        320        330        340        350        360
PGGGSVQIVY KPVDLSKVTS KCGSLGNIHH KPGGGQVEVK SEKLDFKDRV QSKIGSLDNI

        370        380        390        400        410        420
THVPGGGNKK IETHKLTFRE NAKAKTDHGA EIVYKSPVVS GDTSPRHLSN VSSTGSIDMV

        430        440
DSPQLATLAD EVSASLAKQG L
```

Residue	Total number of peptides detected containing a particular residue	Number of peptides detected containing a particular phosphorylated residue	% of phosphorylation
T71	17	1	5.88
T175	8	2	25.00
T181	5	2	40.00
S199	9	1	11.11
T205	9	1	11.11
S208	9	1	11.11
S210	8	3	37.50
T212	49	1	2.04
S214	49	25	51.02
T217	49	1	2.04
T231	2	1	50.00
S262	3	3	100.00
S289	14	2	14.29
S305	12	4	33.33
S324	1	1	100.00
S356	16	10	62.50
S396	5	1	20.00
